# High heterogeneity of cross-reactive immunoglobulins in multiple sclerosis presumes combining of B-cell epitopes for diagnostics: a case-control study

**DOI:** 10.3389/fimmu.2024.1401156

**Published:** 2024-11-28

**Authors:** Leyla A. Ovchinnikova, Igor E. Eliseev, Samir S. Dzhelad, Taras O. Simaniv, Ksenia M. Klimina, Maria Ivanova, Elena N. Ilina, Maria N. Zakharova, Sergey N. Illarioshkin, Yury P. Rubtsov, Alexander G. Gabibov, Yakov A. Lomakin

**Affiliations:** ^1^ Shemyakin-Ovchinnikov Institute of Bioorganic Chemistry, Russian Academy of Sciences, Moscow, Russia; ^2^ St. Petersburg School of Physics, Mathematics, and Computer Science, HSE University, Saint Petersburg, Russia; ^3^ Research Center of Neurology, Moscow, Russia; ^4^ Lopukhin Federal Research and Clinical Center of Physical-Chemical Medicine of Federal Medical Biological Agency, Moscow, Russia; ^5^ Faculty of Biology and Biotechnology, HSE University, Moscow, Russia; ^6^ Faculty of Medicine, Lomonosov Moscow State University, Moscow, Russia

**Keywords:** multiple sclerosis, SPTAN1, Epstein-Barr virus, LMP1, autoantibody, autoantigen, PhIP-Seq, IgG

## Abstract

**Background:**

Multiple sclerosis (MS) is a neuroinflammatory disease triggered by a combination of genetic traits and external factors. Autoimmune nature of MS is proven by the identification of pathogenic T cells, but the role of autoantibody-producing B cells is less clear. A comprehensive understanding of the development of neuroinflammation and the identification of targeted autoantigens are crucial for timely diagnosis and appropriate treatment.

**Methods:**

An expression library of 44-mer overlapping peptides from a panel of putative autoantigenic human proteins was employed for modified Phage ImmunoPrecipitation Sequencing (PhIP-Seq) to identify B cell peptide epitopes from MS patients. Individual peptides extracted by PhIP-Seq were tested by ELISA to characterize their affinity towards IgG from both MS patients and healthy donors (HD). Three candidate auto-peptides were used for isolating autoreactive antigen-specific IgGs from the serum of MS patients.

**Results:**

Autoantibody screening revealed high heterogeneity of IgG response in MS. The autoantigenic genesis of the PhIP-Seq-identified peptides was further strengthened by clinical ELISA testing of 11 HD and 16 MS donors. Validation experiments on independent cohorts of 22 HD and 28 MS patients confirmed statistically significant elevated titers of IgG specific to spectrin alpha chain (SPTAN1) in the serum of MS patients compared to HD. The levels of anti-SPTAN1 IgG correlated in serum and cerebrospinal fluid (CSF). Isolated autoreactive antigen-specific IgG exhibited increased cross-reactivity to a panel of PhIP-Seq-identified antigenic peptides. Serum IgG from MS patients were reactive to latent membrane protein (LMP1) of Epstein-Barr virus, a potential trigger of MS. Discovered antigenic peptides from SPTAN1, protein-tyrosine kinase 6 (PTK6), periaxin (PRX), and LMP1 were tested as potential biomarker panel for MS diagnostics. We concluded that the combination of particular peptides from SPTAN1, PTK6, PRX and LMP1 could be implemented as a four-peptide biomarker panel for MS diagnosis (area under the curve (AUC) of 0.818 for discriminating between HD and MS).

**Conclusions:**

This study supports the concept that the specificity of autoreactive IgG in MS is highly heterogeneous. Despite that we suggest that the combination of several B-cell epitopes could be employed as reliable and simple test for MS diagnostics.

## 1 Introduction

Multiple sclerosis (MS) is an inflammatory autoimmune disease of the CNS resulting in neuronal degeneration leading to severe disability (1). MS has been mostly associated with the T-cell response (2). Currently, it has been established that B cells and their interactions with T cells have significant impact on demyelination in the CNS lesions of MS patients (3). Clonally expanded inflammatory B cells produce characteristic oligoclonal immunoglobulin bands; they shuttle between the bloodstream and the CNS and can be activated in either compartment (4). Autoreactive antibodies directly mediate demyelination and axonal injury both *in vitro* (5) and *in vivo* in animal models (6). B cells also play an important role in the pathogenic presentation of antigens to autoreactive T cells (7). Along with antigen presentation and the production of autoreactive antibodies, the regulatory function of B cells may contribute greatly to the control of inflammation in MS (8–10).

MS is a disease with remarkably heterogeneous immunological outcomes and can be difficult to diagnose at an early stage. Therefore, identifying the target autoantigens that induce autoimmune inflammation is instrumental for both fundamental science and clinical diagnosis. Potential MS biomarkers, represented by characteristic proteins (11) and autoantibodies (12), have already been discovered, but the versatility of these markers is highly controversial. Even anti-myelin antibodies, such as anti-MBP (myelin basic protein), anti-MOG (myelin oligodendrocyte glycoprotein), and anti-PLP (myelin proteolipid protein), are not consistently present in MS patients and can be found in healthy individuals (13).

Historically, MS progression has been associated with various viral infections such as infection with HERV-W endogenous retroviruses (14), varicella zoster (15), human herpesvirus 6 (16), and Epstein-Barr virus (EBV) (17–19). Nevertheless, a direct correlation was firmly established only between EBV infection and MS onset (20). To date, it is well accepted that viral involvement in MS development is implemented through the mechanisms of molecular mimicry and cross-reactivity (21). The Epstein-Barr virus nuclear antigen 1 (EBNA1) and MBP can be cross-recognized by the same T-cell receptor from an MS patient (18). Approximately one-quarter of MS patients were shown to have EBNA1 – GlialCAM (a glial cell adhesion molecule) (12), or EBNA1 – CRYAB (alpha-crystallin B) cross-reactive antibodies (22), while immunizing a mouse with the EBNA-1 promotes CNS inflammation in EAE (experimental autoimmune encephalomyelitis) animal models. Similarly, we have previously shown that exposure to another EBV antigen, latent membrane protein 1 (LMP1), induced the production of autoreactive anti-MBP antibodies in mice (23). Moreover, therapy with autologous EBV-specific T cells targeting LMP1, LMP2A and EBNA1 improves the clinical outcome of patients with progressive MS (24). However, the exact fingerprint of antibodies against viral antigens following MS development has not been defined yet.

Identifying a heterogeneous array of autoimmune targets in MS remains a challenge. The combination of phage display technology with subsequent sequencing (25–27), high-precision protein microarrays (28), and immunoprecipitation followed by mass spectrometry (29) can be used to discover antibody targets. We generated the fd phage library containing a panel of overlapping peptide fragments that tile across human proteins most likely involved in autoimmune pathologies. In-depth antibody profiling using purified serum IgG from patients with stable multiple sclerosis (SMS) and active multiple sclerosis (AMS) was performed. Our results demonstrate high heterogeneity of autoimmune targets in the sera of MS patients and reveal novel possible links between autoimmune aggression during MS and disease severity. Combinations of peptide autoantigens identified by PhIP-Seq screening could be employed for the diagnosis of MS and pretreatment stratification of the patients.

## 2 Materials and methods

### 2.1 Study design, patient and donor information

**Figure 1:**
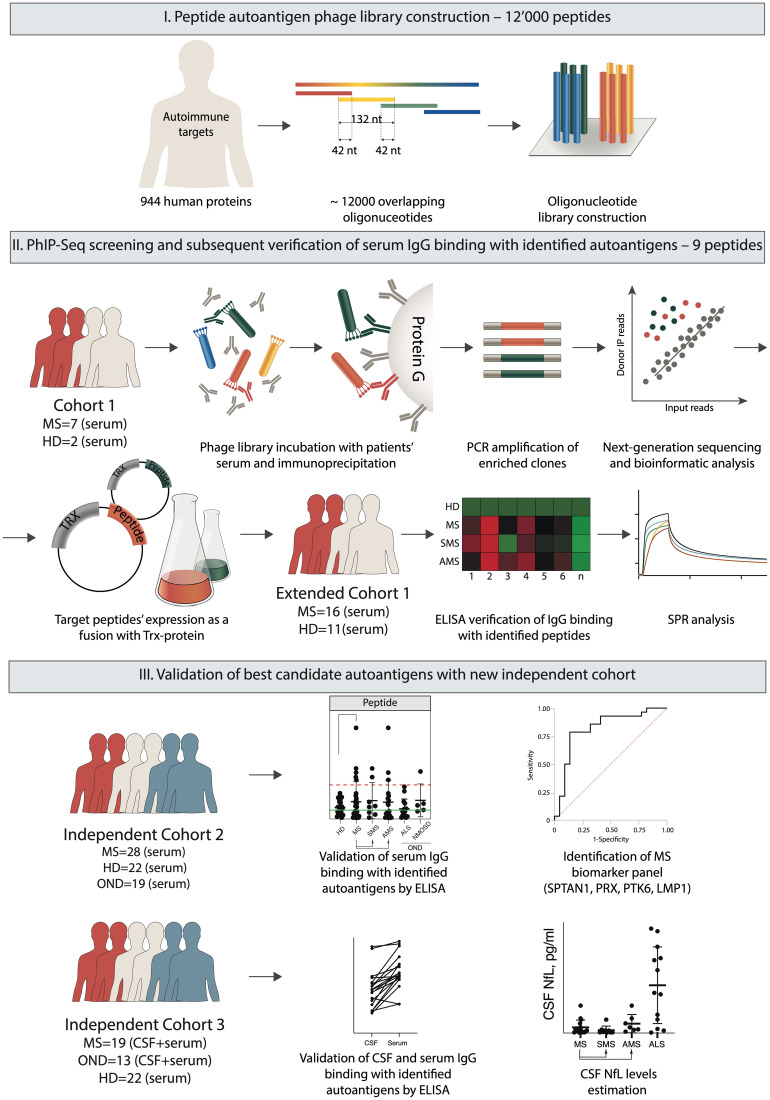
Overview of the experimental design. The autoantigen peptide library was constructed to display 44 amino acid overlapping peptides on the surface of fd bacteriophage particles. Serum IgG samples from MS patients and HD were immunoprecipitated with a phage library. Bound bacteriophages were eluted from Protein G, and peptide-encoding sequences were amplified for subsequent NGS. Based on the NGS data, the identified sequences encoding the target candidate autoimmune antigens were expressed in a prokaryotic system fused with the TRX carrier protein for subsequent antigen identification by ELISA and SPR. Finally, the best candidate autoantigens were validated by ELISA using a set of sera and/or CSF from independent cohorts of HD and patients with MS and OND.

The study was composed of the following three phases: (*i*) peptide autoantigen phage library construction, (*ii*) screening and extraction of peptide autoantigens, and (*iii*) validation of the best candidate autoantigens. The last step required testing of serum IgG from independent cohorts of HD, patients with MS and other neurological diseases (OND) for binding with immobilized candidate antigens by ELISA (**Figure 1**). Finally, the reactivity of IgG to the selected peptides was evaluated using cerebrospinal fluid (CSF) from patients with MS.

Blood samples from the initial cohorts of 11 healthy donors (HD) and 16 MS patients (**Supplementary Table S1**) were collected in 2020 at the Research Center of Neurology (Moscow) and were used in antigen screening experiments. A total of 9 serum samples were subjected to PhIP-Seq. Later, titers of IgGs that bind identified MS-associated peptides were defined in all 27 samples of donor serum by ELISA. The antigenic peptides extracted from the peptide library were validated by ELISA of serum IgG samples from independent cohorts of 22 HD and 28 MS (**Supplementary Table S2**), blood samples were collected in 2023 at the Research Center of Neurology (Moscow). The serum of OND patients [14 with amyotrophic lateral sclerosis (ALS) and 5 with neuromyelitis optica spectrum disorder (NMOSD)] served as negative controls in the validation experiments (**Supplementary Table S2**). An additional cohort of 19 patients with MS and 13 patients with ALS was included in 2024 for analysis of CSF and paired serum samples (**Supplementary Table S3**). CSF was collected from donors who underwent lumbar puncture during a routine diagnostic examination to confirm neurological condition. Blood and CSF samples were collected and then centrifuged at 4°C (blood: 1500 g, 10 min; CSF: 1000 g, 10 min). Supernatants were immediately frozen, stored at –80°C and shipped on dry ice for further analysis. MS was diagnosed according to the McDonald criteria. Relapsing-remitting active MS was diagnosed based on two or more relapses with incomplete recovery during one year, and high MRI activity (two or more newly developed gadolinium-enhanced (GD+) brain lesions during one year). Relapsing-remitting stable MS was diagnosed if the EDSS score was below 5.0 with no exacerbations, no increase in the EDSS score, and the absence of cerebral enhancing lesion on MRI in the preceding two years. Inclusion criteria for AQP4 seropositive NMOSD were based on the 2015 International Panel on NMOSD Diagnosis which requires AQP4 antibody seropositivity tested by cell-based assay and inflammatory attack(s) of the optic nerve, spinal cord, or brain, including the brainstem and area postrema attacks (30). Patients were diagnosed with clinically definite ALS according to the El Escorial criteria which requires the evidence of lower motor neuron degeneration by clinical and electrophysiological examination, evidence of upper motor neuron degeneration by clinical examination, and progressive spread of symptoms or signs within a region or to other regions, as determined by history or examination (31).

### 2.2 Computational autoantigen library design and cloning

Unbiased design would give enormous number of random peptides; therefore, the complexity of the peptide library was artificially limited. To identify novel IgG autoantigens in MS, we selected human targets among the proteins associated with antibody response during various autoimmune disorders [AAgAtlas project - http://biokb.ncpsb.org/aagatlas (32, 33)], but not previously described as established autoantigens during MS. We also included several CNS-associated proteins involved in neuronal survival, differentiation, or growth (34–36), that had not been previously linked to MS progression. A total of 11,973 unique peptides from 302 (6424 peptides) and 642 (5536 peptides) human proteins associated with various autoimmune pathologies and cellular protein degradation pathways, respectively, were included (**Supplementary Table S4**). Each protein sequence was split into 44 amino acid peptides with 14 amino acid overlaps. The resulting peptides were computationally reverse translated into 132 nt coding sequences, which were subsequently optimized for prokaryotic expression. For PCR amplification, 17 nt (CCCAGCCGGCCATGGCC) and 20 nt (GCTAGCAGTGGTGGAGGCGG) adapters were added to the 5′ and 3′ ends of each sequence. All formed internal restriction sites NcoI/NheI were changed to alternative codons encoding the same amino acids. The corresponding oligonucleotides were chemically synthesized (Twist Bioscience). To achieve the uniform distribution of peptide sequences and efficient DNA amplification, the final autoantigen library was amplified using emulsion PCR (37). The amplified library was cloned into the fd bacteriophage vector to produce peptide in-frame fusions with the bacteriophage coat protein p3 (fADL-1e-based vector AddgeneID139441) (27, 38) and transformed into *E. coli* TG-1 strain. Resulting autoantigen library had 94% coverage of unique sequences, as determined by NGS at a mean mapped read depth of 32x. Unique peptide sequences in the designed library were detected with a relatively uniform distribution (**Supplementary Figure S1**). Phage virions were obtained using the established protocol (38, 39). Briefly, recombinant bacteriophages were purified by double PEG precipitation. The titer of the phage library was 5·10^13^ PFU/ml. The concentration of the bacteriophage particles was determined by ELISA. Purified phage particles were captured on the immunosorbent plate (Nunc) with immobilized anti-M13 antibodies (Sigma-Aldrich) and detected with horseradish peroxidase (HRP) conjugated anti-M13 (GE Healthcare) and anti-FLAG antibodies (Sigma-Aldrich). The resulting phage library was aliquoted and stored at -80°C until further use.

### 2.3 IgG purification and Phage ImmunoPrecipitation Sequencing

IgGs were isolated from serum by double-repeated 50% ammonium sulfate precipitation, followed by affinity chromatography on protein G-Sepharose (Amersham Biosciences, UK) and size-exclusion chromatography on Superdex 200 (GE Healthcare, UK). The standard protein G PhIP-Seq protocol has been described previously (40). Briefly, 1 mL of phage library (5·10^9^ PFU) was incubated with purified human serum IgG (2 µg) for 18 h at 4°C with rotation. Next day, 10 µL of protein G-coated magnetic beads (Invitrogen) were mixed with each sample and incubated for 4 h at 4°C with rotation. The beads were washed with PhIP-Seq wash buffer (50 mM Tris-HCl, pH 7.5, 150 mM NaCl, 0.1% NP-40) three times, and the bound phages were lysed at 95 °C for 10 min in water. The initial phage library without immunoprecipitation and mock immunoprecipitates (beads used on the phage library without serum pre-incubation) were used as controls. The final immunoprecipitated phage lysates were used for the preparation of the NGS library. To achieve the uniform distribution of enriched peptide sequences and efficient DNA amplification, we utilized emulsion PCR for DNA amplification as described previously (37) with primers flanking the designed peptide inserts. Illumina sequencing sample indexes and adapters were added within subsequent PCR.

Paired-end libraries were prepared with NEBNext Ultra II DNA Library Prep Kit (New England Biolabs, USA) in accordance with the manufacturer’s recommendations. The libraries were indexed with NEBNext Multiplex Oligos kit for Illumina (96 Index Primers, New England Biolabs, USA). Size distribution of the libraries and their quality were assessed using a high-sensitivity DNA chip (Agilent Technologies, USA). Subsequently, the libraries were quantified by High Sensitivity Quant-iT DNA Assay Kit (Thermo Scientific, USA). DNA sequencing was performed on the MiSeq platform (Illumina, USA) according to the manufacturer’s recommendations using the MiSeq Reagent Kit v2 (300 cycles) and a 10% PhiX spike-in control.

Linked 5′ and 3′ adapters in the NGS reads were identified and trimmed with *cutadapt* (41). Only reads with both adapters and no insertions or deletions in both paired-end reads were used for further analysis. Trimmed reads were aligned to the oligo library with *bowtie* (42), allowing for a maximum of three mismatches. Read counts were extracted using *phip-stat* (40) and normalized across all samples via the size-factors method (43). For each peptide, we computed a z-score using a statistical procedure proposed by Mina et al. (44) and implemented in *phippery* (https://github.com/matsengrp/phippery). Three mock immunoprecipitations were used for binning. Each bin was composed of minimum of 300 peptides that had similar counts in the mock immunoprecipitations. When analyzing each immunoprecipitation, the peptides that fell within the middle 90% according to the number of counts (a subset assumed not to contain hits) were used to estimate the mean and standard deviation of the peptide counts after immunoprecipitation within each bin. These estimates were then used to calculate the z-score for all peptides from that bin. Standardized enrichments were computed with *phippery*, and peptides with z-score ≥ 3 and standardized enrichment ≥ 2 were selected. Peptides were considered reactive if the counts exceeded 400 in at least two patients in addition to fulfilling the z-score and enrichment criteria.

### 2.4 Protein expression and purification

Peptide-TRX-coding plasmids were generated by PCR amplification of the coding sequence from the enriched libraries with a set of specific primers (**Supplementary Table S5**) and transferred into the pET-TRX-6xHis vector. Protein expression of the TRX-fused peptides was performed in the *E. coli* cell strain BL21 DE3. The overnight culture was inoculated at a 1:100 (*v/v*) ratio into 2xYT media supplemented with ampicillin (100 μg/mL) and 0.1% glucose. Cell culture was grown at 37°C to an OD600 of 0.4–0.6 and then supplemented with 0.5 mM of the IPTG (isopropylthio-β-galactoside). The expression culture was grown for 4 h at 30 °C with intensive aeration. Collected pellets were lysed with 0.2 mg/mL of lysozyme in PBS supplemented with cOmplete protease inhibitor cocktail (Roche, Germany). Lysates were incubated on ice with 10 μg/mL DNase until the viscosity disappeared. The resulting solution was centrifuged at 20,000 g and 4°C for 20 minutes, filtered through a 0.22 μm membrane and purified utilizing Ni-NTA resin according to the manufacturer’s instructions (Qiagen, USA). 6xHis-tagged peptides fused with TRX were then purified on Superdex 75 size-exclusion column. Recombinant intracellular LMP1 fragments (188-263 a.a.; 257-330 a.a.; 325-386 a.a.) were obtained in bacterial cells, while full-length LMP1 was produced in mammalian cells (19).

### 2.5 Quantitative measurement of antigen-specific IgGs in human serum and CSF by ELISA

The antigens, diluted with 100 mM carbonate buffer (pH 9.0) to a concentration of 5 μg/mL, were absorbed on 96-well plates (MaxiSorb) overnight at 4°C and blocked with 2% nonfat dry milk in carbonate buffer. Starting from the 200 µg/mL dilution, purified serum antibodies were serially diluted at a 1:2 or 1:5 ratio in PBS with 0.5% nonfat dry milk and 0.05% Tween 20, applied to a coated well, and incubated for 1 h at 37°C with rotation. CSF samples were diluted at a 1:1 ratio in the same buffer. For isolated antigen-specific IgG, a concentration of 40 ng/mL was applied. Serum and isolated antigen-specific IgG samples were assayed in triplicate, while CSF samples were assayed in duplicate. As all recombinant antigens contained 6xHis tag, titration of anti-His antibodies (Sigma-Aldrich, USA) was used for calibration. After three washes with PBS containing 0.1% Tween 20, bound antibodies were detected with HRP-conjugated anti-human IgG Fc-specific antibodies (1:5,000) (ThermoFisher Scientific, USA). After 1 h at 37°C with rotation, plates were washed 5 times, and TMB was added for 15 min. The reaction was stopped with 10% H_3_PO_4_. The OD450 values were measured on the Varioskan Lux microplate reader (Thermo Scientific, USA).

### 2.6 Surface plasmon resonance-based binding analysis of purified human serum IgG

Surface plasmon resonance (SPR) measurements were performed using Biacore T200 (GE Healthcare, USA). Molecular interactions were registered as sensograms representing the records of biosensor signals [in resonance units (RU)] as time function. Using the Biacore amine coupling kit, SPTAN1_601-644_ fused with TRX and a reference TRX-carrier without peptide were immobilized in the flow cells 3 and 2 of the CM5 sensor chip, respectively, following the standard protocol. The entire coupling procedure resulted in 800 RU of immobilized proteins. Flow cell 1 with blank immobilization was used as an additional negative control. To evaluate the affinity of purified serum IgG, each patient’s sample was used in concentration 1 mg/mL in HBS-EP buffer with the addition of HSA to 5 mg/mL to eliminate nonspecific binding. Each sample was injected over 500 s, and then dissociation curve in a running buffer was measured over 2000 s. All SPR experiments were performed in triplicate. Assays were carried out at 20°C with a flow rate of 15 µL/min. The association (k_a_) and dissociation rate constants (k_d_) were calculated from the sensograms using two-state kinetic models in *Prism*.

### 2.7 Isolation of antigen-specific autoantibodies from serum IgG of MS patients

To isolate antigen-specific autoantibodies, a preliminarily step involving the depletion of non-specific antibodies was carried out. For this purpose, 15 mg of purified IgG from serum of MS donors were incubated with 5 µL of NHS Mag Sepharose (GE Healthcare, USA) conjugated with TRX-carrier protein in TBS buffer (150 mM NaCl, 50 mM Tris-HCl, pH 7.5) at room temperature with rotation for 1 hour. TRX-specific and non-specifically bound antibodies were then removed from the solution together with NHS-TRX magnetic beads. Next, flow-through from depletion step was incubated with 5 µL of NHS Mag Sepharose conjugated with one of the recombinant peptide antigens fused with TRX-carrier: KRT1_301-344_-TRX, SPTAN1_601-644_-TRX, or GPI_31-74_-TRX. The antibodies were incubated with appropriate antigen-TRX magnetic beads for 1 h at room temperature with rotation. Next, peptide-coupled magnetic Sepharose beads were washed twice in TBS, followed by washing with 50 mM sodium acetate buffer, pH 5.0 to remove non-specific antibodies. Antigen-specific antibodies were then eluted using 100 mM glycine-HCl buffer, pH 2.0, and the pH of the eluate was immediately adjusted to 7.0 using 1 M Tris-HCl buffer to prevent conformational changes of the eluted antibodies. The isolated antigen-specific antibodies were further analyzed using ELISA and polyacrylamide gel electrophoresis (PAGE).

### 2.8 Identification of common motifs in the analyzed peptides

The search for motifs in the analyzed peptides was conducted with the MEME (Multiple Em for Motif Elicitation) tool used in the Classic Discovery mode (http://meme-suite.org/tools/meme). The motifs with E-value < 0.05 were considered significant according to the MEME algorithm guidelines.

### 2.9 ROC analysis

The CombiROC method was used to determine the diagnostic accuracy of optimal combinations of serum autoreactive IgG levels (45). This enabled determining the best combinations of biomarkers by a combined analysis of receiver operating characteristic (ROC) curves, calculating the sensitivity (SE) and specificity (SP) of all possible marker combinations (http://CombiROC.eu).

### 2.10 CSF neurofilament light chain measurements

CSF neurofilament light chain (NfL) levels were determined by ELISA using commercially available monoclonal antibodies against human NfL (NF79 and HRP-conjugated NF71, HyTest, Russia) and recombinant NfL standard (RPE038Hu03, Cloud-Clone Corp., Wuhan, China). Measurements with a blank sample and NfL standard were used for signal calibration. Samples were run simultaneously in duplicates. CSF samples were standardly diluted at 1:4 ratio.

### 2.11 Statistical analysis

Data was analyzed using GraphPad Prism, version 9. The level of significance for the statistical analyses was set at p < 0.05. The antibody index for each sample was calculated as follows: (sample ELISA OD_450_ value – mean blank value)/(mean HD ELISA OD_450_ value – mean blank value). The statistical significance of differences in categorical data (e.g., seroprevalence) was assessed using Fisher’s exact test, as appropriate for relatively small sample sizes. In ELISA assays, samples were defined as seropositive if signals exceeded the mean signal for the HD group by three standard deviations. Several measurements within the HD group were identified as outliers by Grubbs’ test and were subsequently excluded from the cut-off calculation. Then, contingency tables containing the number of hits above this cut-off were analyzed to calculate Fisher’s exact p-values. The Mann–Whitney U test was used to evaluate the statistical significance of differences in ELISA signals between independent samples. Correlations between continuous variables were tested with the Pearson correlation coefficient.

### 2.12 Standard protocol approvals, registrations, and patient consents

The study was approved by the Local Ethic Committee of the Research Center of Neurology, Moscow, Russia. All patients and HD provided informed consent before participation.

## 3 Results

### 3.1 Identification and verification of candidate MS antigens from PhIP-Seq data

**Figure 2:**
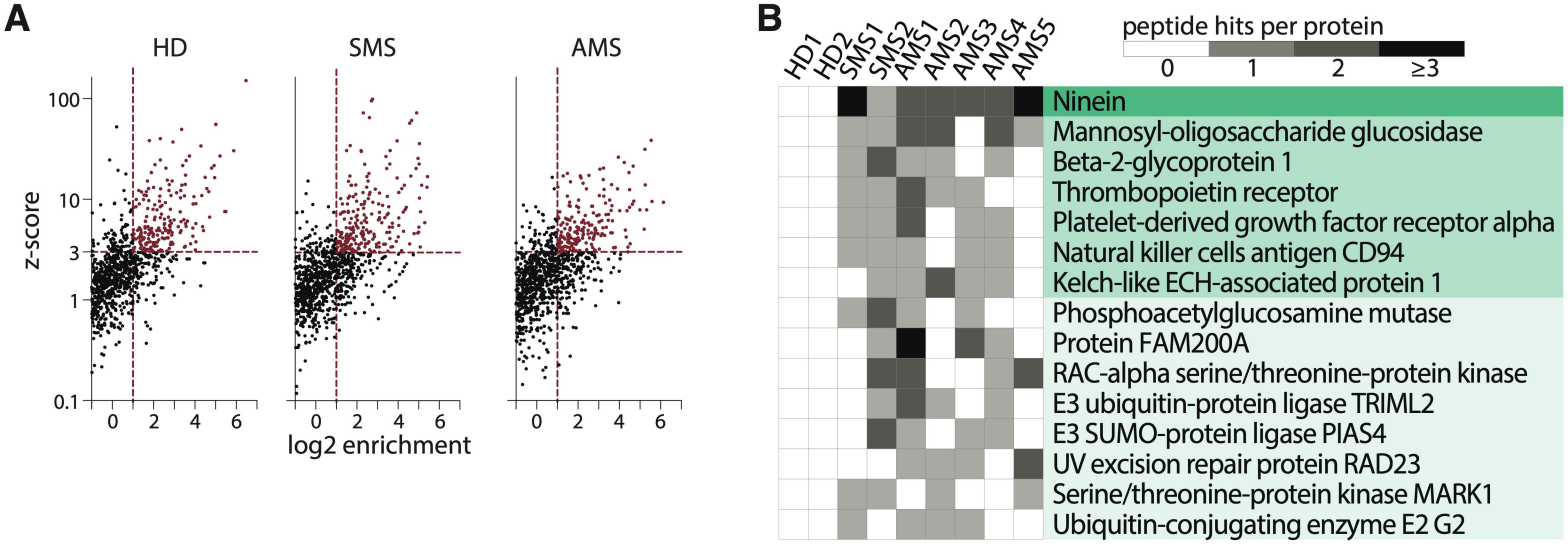
Autoantigen peptides and proteins identified by PhIP-Seq. **(A)** The scatter plots illustrate a representative analysis of the enrichment of the human peptidome library for healthy donors (HD), patients with stable multiple sclerosis (SMS), or active multiple sclerosis (AMS) according to z-score and enrichment criteria. **(B)** A heatmap showing the distribution of seroactive MS-associated peptides from the corresponding proteins. Peptide hits per protein indicate the number of distinct enriched peptides constituting the designated whole protein. Each column represents one individual.

The phage library containing overlapping unique 44-amino acid peptides covering human proteins associated with autoimmune disorders was incubated with serums from patients with two types of MS (stable MS, SMS; and active MS, AMS) or HD (**Supplementary Table S1**). Characteristics of patients in the MS and HD cohorts are summarized in **Supplementary Tables S1**, **S2**. After immunoprecipitation with protein G, the enriched peptides were identified via NGS analysis. The number of enriched peptides did not significantly differ between HD and both MS cohorts (mean ± standard deviation of enriched peptides in HD: 181 ± 30; SMS: 164 ± 82; AMS: 143 ± 28) (**Figure 2A**; **Supplementary Figure S2**). To analyze whether different individuals or even the same donor produce antibodies recognizing distinct epitopes of the same protein, the extracted peptides were juxtaposed against the ORF enrichment matrix. The largest number of individual peptides seroactive for all tested MS patients was detected in ninein (**Figure 2B**). Several peptides from mannosyl-oligosaccharide glucosidase (MOGS) were found in the serum of most MS patients but not in HD cohort. While antibodies reactive for most proteins recognized single 44-mer peptides, reactivity to ninein and MOGS among MS patients was detected for several peptides from different parts of the proteins. This indicates that these autoantibodies may bind many epitopes from one polypeptide, suggesting a polyclonal, antigen-driven response during neuroinflammation (**Figure 2B**; **Supplementary Figure S4**).

The peptides ranking was done according to their enrichment in each sample. Significant enrichment of 8 potential autoantigens was found in MS patients but not in HD. A set of peptides (SPTAN1_601-644_, DPP4_481-524_, GPI_31-74_, HAVCR1_31-74_, POU4F1_91-134_) that had a z-score > 3 in at least three MS patients, but had a z-score < 3 in all healthy donors was identified, and these peptides were on the top of the hit list (UniProt abbreviations of gene names are used). Three peptides (PRX_451-494_, PTK6_301-344_, KRT1_301-344_) with lower enrichment in the MS group but non-immunoreactive (z-score < 0) in healthy donors were also included. The peptide INSR_301-344_, which was enriched in both MS and HD groups, was added to the list too. The antigenic peptides selected for further analysis are shown in **Table 1**. Corresponding proteins have not yet been linked with MS.

**Table 1:** Autoimmune targets potentially involved in MS.

AA	UniProt	Gene symbol	Protein name
LQAKLDNLQQEIDFLTALYQAELSQMQTQISETNVILSMDNNRS	P04264	KRT1	Keratin, type II cytoskeletal 1
YKDPSNLQGKVQKHQAFEAELSANQSRIDALEKAGQKLIDVNHY	Q13813-3	SPTAN1	Spectrin alpha chain non-erythrocytic 1, Isoform 3
TLHSSVNDKGLRVLEDNSALDKMLQNVQMPSKKLDFIILNETKF	P27487	DPP4	Dipeptidyl peptidase 4
KLPKVPEAALPEVRLPEVELPKVSEMKLPKVPEMAVPEVRLPEV	Q9BXM0	PRX	Periaxin
DANKDRFNHFSLTLNTNHGHILVDYSKNLVTEDVMRMLVDLAKS	P06744	GPI	Glucose-6-phosphate isomerase
SVTLPCHYSGAVTSMCWNRGSCSLFTCQNGIVWTNGTHVTYRKD	Q96D42	HAVCR1	Hepatitis A virus cellular receptor 1
CHQYVIHNNKCIPECPSGYTMNSSNLLCTPCLGPCPKVCHLLEG	P06213	INSR	Insulin receptor
TSTSTVPLAHHHHHHHHHQALEPGDLLDHISSPSLALMAGAGGA	Q01851	POU4F1	POU domain, class 4, transcription factor 1
CYLESQNYIHRDLAARNILVGENTLCKVGDFGLARLIKEDVYLS	Q13882	PTK6	Protein-tyrosine kinase 6

**Figure 3:**
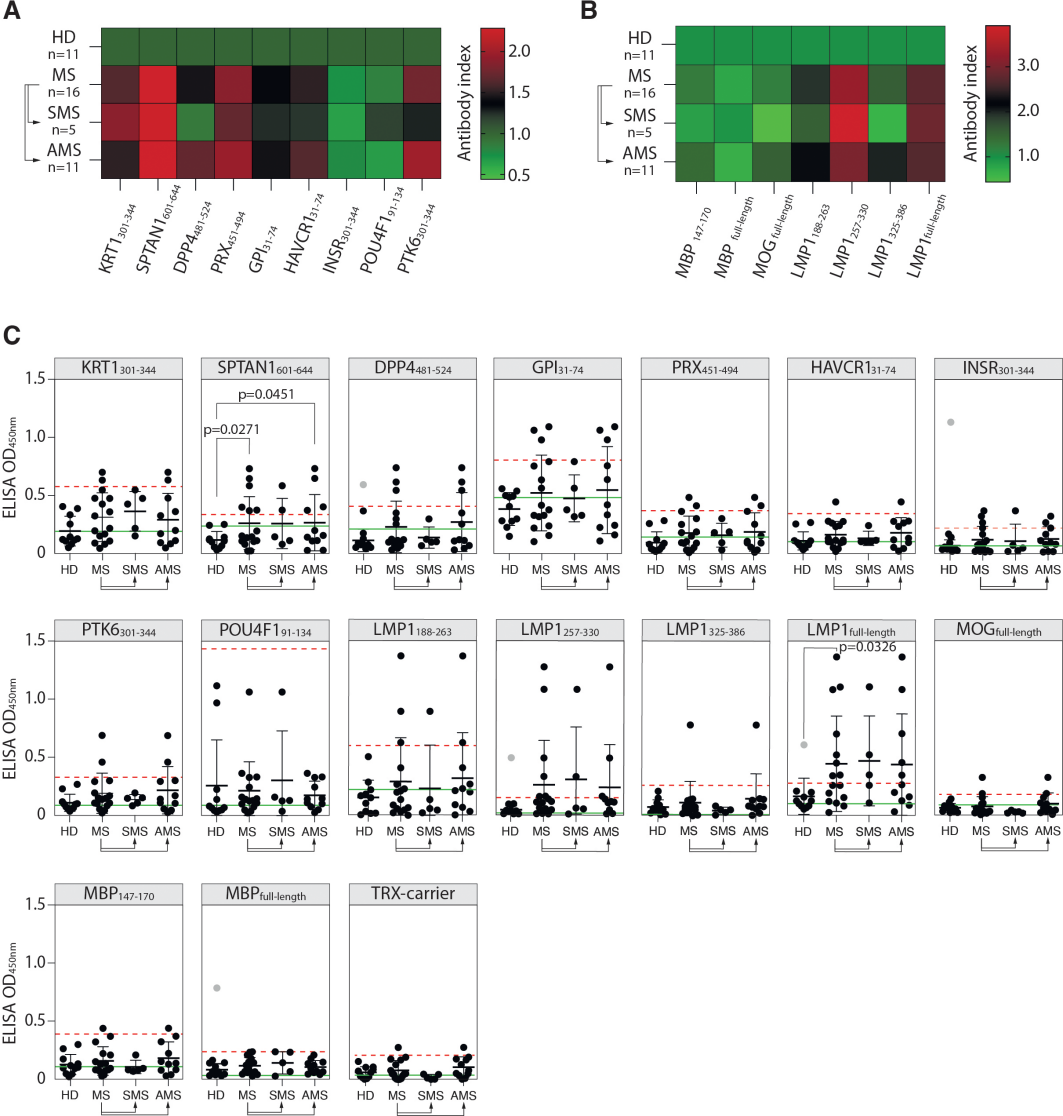
Reactivity of MS patients’ serum IgG towards PhIP-Seq-identified antigens. **(A)** Mean binding of serum IgG from MS patients and HD with peptides identified by PhIP-Seq; or **(B)** with myelin antigens (MBP, MOG, immunodominant MBP_147-170_ fragment) and EBV antigens (full-length LMP1 and LMP1 C-terminal peptides: a.a. residues 188-263, 257-330, and 325-386), assessed by ELISA and presented as heatmaps. **(C)** ELISA verification of binding activity of serum antibodies from MS patients and HD with identified PhIP-Seq-selected peptides, full-length EBV LMP1 and its C-terminal fragments 188-263, 257-330, 325-386 a.a., full-length MPB and its 147-170 a. a. fragment displayed as dot diagrams. Each dot represents an individual donor. Mean values ± SD (error bars) are shown. Dashed red line indicates the mean of healthy control signal + 3 standard deviations. Outliers within the HD group that were excluded from cut-off calculation are shown in gray. Green line – negative control representing binding of IVIG (pooled IgG from 1000 healthy donors) for each peptide. Fisher’s exact test was used to determine the statistical significance of the differences in the number of hits that exceeded the three standard deviation cut-offs between donor groups. Only p-values less than 0.05 are indicated to show statistically significant differences.

The reactivity of MS and HD serum antibodies to the identified autoreactive peptides was verified by ELISA. Purified total IgG from serums of 16 MS patients and 11 HD were used for ELISA with identified peptides fused to TRX (**Table 1**). Elevated binding with serum IgG was observed for seven of the nine peptides in MS group, except insulin receptor (INSR_301-344_) and transcription factor 1 (POU4F1_91-134_), but none in HD samples (**Figures 3A, C**). Although there was a marked increase in mean binding for the majority of the analyzed peptides in MS samples, statistically significant difference in the number of seropositive samples between MS and HD groups was detected only in case of SPTAN1_601-644_. Individuals with significantly elevated titers of antibodies against SPTAN1_601-644_ were present in both SMS and AMS groups. Antibodies against KRT1_301-344_, DPP4_481-524_, PRX_451-494_, HAVCR1_31-74_, PTK6_301-344_, and GPI_31-74_ were predominantly observed in AMS patients. No difference was found between HD and MS patients for the binding of serum antibodies with well-characterized myelin antigens, such as MBP and MOG (**Figures 3B, C**).

### 3.2 Reactivity of serums from MS donors with peptides from EBV LMP1 and PhIP-selected peptides

Assuming the association of EBV with MS (12, 20), we studied whether MS patients had elevated titers of serum antibodies against the EBV antigen LMP1, for which cross-reactive anti-LMP1 antibodies associated with MS were earlier reported by our group (19, 23). MS patients had elevated titers of serum antibodies recognizing both the full-length LMP1 and its C-terminal fragments LMP1_188-263_ and LMP1_257-330_ (**Figures 3B, C**). A statistically significant difference in the number of seropositive patients was detected between the HD and MS groups in the case of the full-length LMP1. Although the number of seropositive patients with IgG against LMP1 fragments did not differ significantly between HD and MS groups, most of the patients reactive to these fragments were also seropositive for the full-length LMP1. Increased titers of anti-LMP1 antibodies were also observed in some individuals in the HD group. However, there was no significant correlation between the binding of LMP1/LMP1-derived peptides and PhIP-Seq-selected antigenic peptides with total IgG from MS donors (**Supplementary Figure S4A**). Likewise, no association between the level of LMP1-binding antibodies and EDSS scores was observed (**Supplementary Figure S4B**).

### 3.3 SPR analysis of SPTAN1_601-644_ binding to antibodies from MS donors

**Figure 4:**
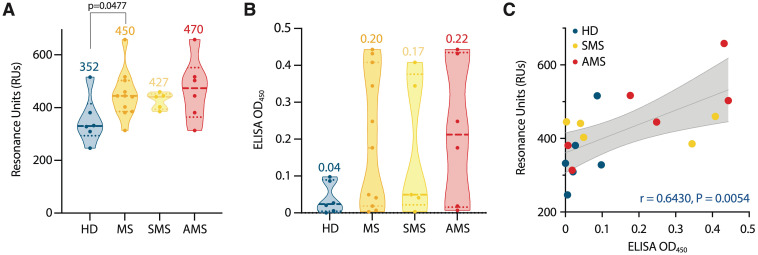
Characterization of serum autoantibody binding. **(A)** Purified serum IgGs from MS patients (MS) and healthy donors (HD) were analyzed for binding with identified SPTAN1_601-644_ auto-peptide by SPR. Total antibody binding is represented in SPR resonance units (RUs). **(B)** SPTAN1_601-644_ auto-peptide binding by corresponding IgGs examined by ELISA. Each dot represents a distinct patient, color coded by groups: blue, healthy donors (n= 6); orange, MS (n= 11); yellow, SMS (n= 5); red, AMS (n= 6). The mean values for each group are shown. **(C)** Correlation and linear regression of SPTAN1_601-644_ peptide binding assessed by ELISA or SPR. The associated Pearson’s correlation coefficients (r) and linear regression significance (P) are indicated. Trend line fit is shown as the center line with the error bands representing the 95% confidence intervals shown as shaded areas. All SPR and ELISA experiments were performed three times, the variation for each sample across triple SPR runs was <5%.

In all samples, the binding of IgG to TRX-SPTAN1_601-644_ was detected (**Supplementary Figure S5**). Stronger binding was observed for serum samples from MS group and, especially, for AMS group (compared with HD) (**Figure 4A**). Although AMS patients had the highest average titers of IgG against TRX-SPTAN1_601-644_, statistically significant difference in maximum resonance units (RUs) signals was observed only when HD group was compared to combined MS group (AMS plus SMS data). This result demonstrates that the size of the cohorts matters. To evaluate the antibody avidity against TRX-SPTAN1_601-644_, the dissociation kinetics (off-rate constants) of the antigen-antibody complexes was assessed, as this kinetics does not depend on the concentration of antigen-specific antibodies (46). All analyzed serum IgG from both HD and MS cohorts had high antibody avidity against SPTAN1_601-644_ with the off-rates ranging from 2·10^-3^ to 6·10^-3^ s^-1^. It should be emphasized that ELISA analysis of IgG binding to TRX-SPTAN1_601-644_ among the same donors demonstrated similar to SPR elevated reactivity for MS patients (**Figure 4B**). Significant positive correlation between the titers of the autoreactive anti-SPTAN1_601-644_ IgG detected by SPR and ELISA in the serum samples from HD and MS patients was observed (according to Spearman test r = 0.6430; *p*= 0.0054) (**Figure 4C**).

### 3.4 Cross-reactivity of autoantibodies from MS donors recognizing KRT1_301-344_, SPTAN1_601-644_, and GPI_31-74_

**Figure 5:**
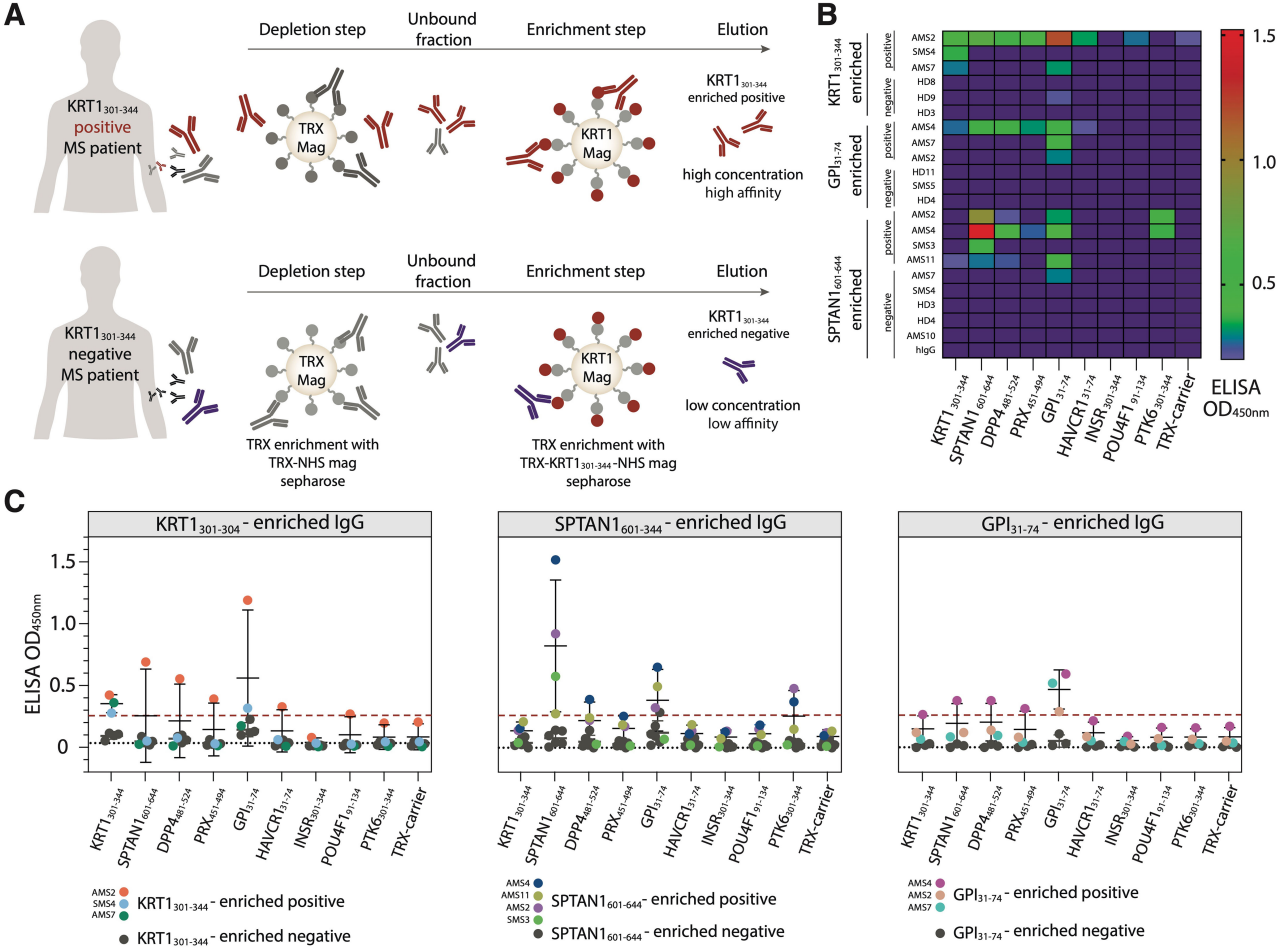
Isolated autoantibodies from the serum of MS patients reveal elevated cross-reactivity with human autoantigens. **(A)** Scheme of the isolation of autoreactive IgG. Purified antibodies from the antigen-positive MS patients (MS patients with increased IgG level against analyzed antigen) were first depleted for anti-TRX antibodies and then enriched for autoantigen-specific antibodies utilizing magnetic beads, coated with the analyzed antigen. After washing and elution steps, the fractions of antigen-enriched positive antibodies were acquired. The same procedure applied to the antigen-negative patients results in antigen-enriched negative antibodies. **(B)** Heatmap showing the binding activity of the SPTAN1_601-644_-, KRT1_301-344_- and GPI_31-74_-specific enriched antibodies. **(C)** Relative binding activity of the antigen-enriched antibodies. Antigen-enriched negative samples are black, individual antigen-enriched positive samples are colored. Dot plots with error bars show mean ± SD. Dashed red line indicates the cut-off OD value. Dashed black line indicates additional negative control binding of IVIG (pooled IgG from 1000 healthy donors).

Next, we wondered whether autoantibodies from peripheral blood of MS donors, which specifically recognize KRT1_301-344_, SPTAN1_601-644_, or GPI_31-74_ peptides, are polyreactive and can cross-react with other peptides. Thus, autoreactive IgG were incubated with magnetic beads coated by the corresponding autoantigens fused to TRX (**Figure 5A**). IgG fractions both from the donors with increased titers of IgG to the particular analyzed antigen and from those without detectable reactivity to the same antigen were studied. To decrease the non-specific binding of TRX with IgG, antibodies specific to the TRX were depleted from the total IgG pool of MS patients’ and HD sera. The enrichment of the IgG pools with antibodies against the PhIP-Seq-identified peptides (SPTAN1_601-644_, KRT1_301-344_ or GPI_31-74_) was carried out using the so-called positive and negative samples from donors reactive and non-reactive respective to the antigenic peptides. As expected, the positive samples enriched with IgG specific to PhIP-Seq-identified peptides had high affinity to the antigen that was initially chosen for enrichment (**Figures 5B, C**). Concentrations of IgG fractions from positive samples eluted from TRX-peptide—coated beads were relatively high (5-30 µg/mL). In contrast, the IgG preparations obtained in parallel from the “negative” samples contained low levels (< 1 µg/mL) of IgG and demonstrated weak or no binding to all antigens identified in this study (**Figures 5B, C**). At the same time, IgG from HD serum samples and human IVIG (additional negative control representing pooled IgG from 1000 HD) didn’t cross-react with the tested antigens. The antigen-positive antibodies from SMS donors (0/2) showed no cross-reactivity to the analyzed antigens, while IgG from AMS donors (6/8) cross-reacted with at least two analyzed antigens (**Figure 5B**). A detailed comparison of antigen-enriched IgG samples revealed significant cross-reactivity of antigen-specific IgG binding SPTAN1_601-644_ and GPI_31-74_ (**Supplementary Figure S6**).

Amino acid sequences of PhIP-Seq-identified peptides showed significant disparity, suggesting a limited level of conservation; only short common motifs for the peptide pairs KRT1 and SPTAN1, GPI and SPTAN1, DPP4 and SPTAN1, KRT1 and GPI were identified (**Supplementary Figure S7**).

### 3.5 Independent validation of the identified antigens and their significance as clinical biomarkers

**Figure 6:**
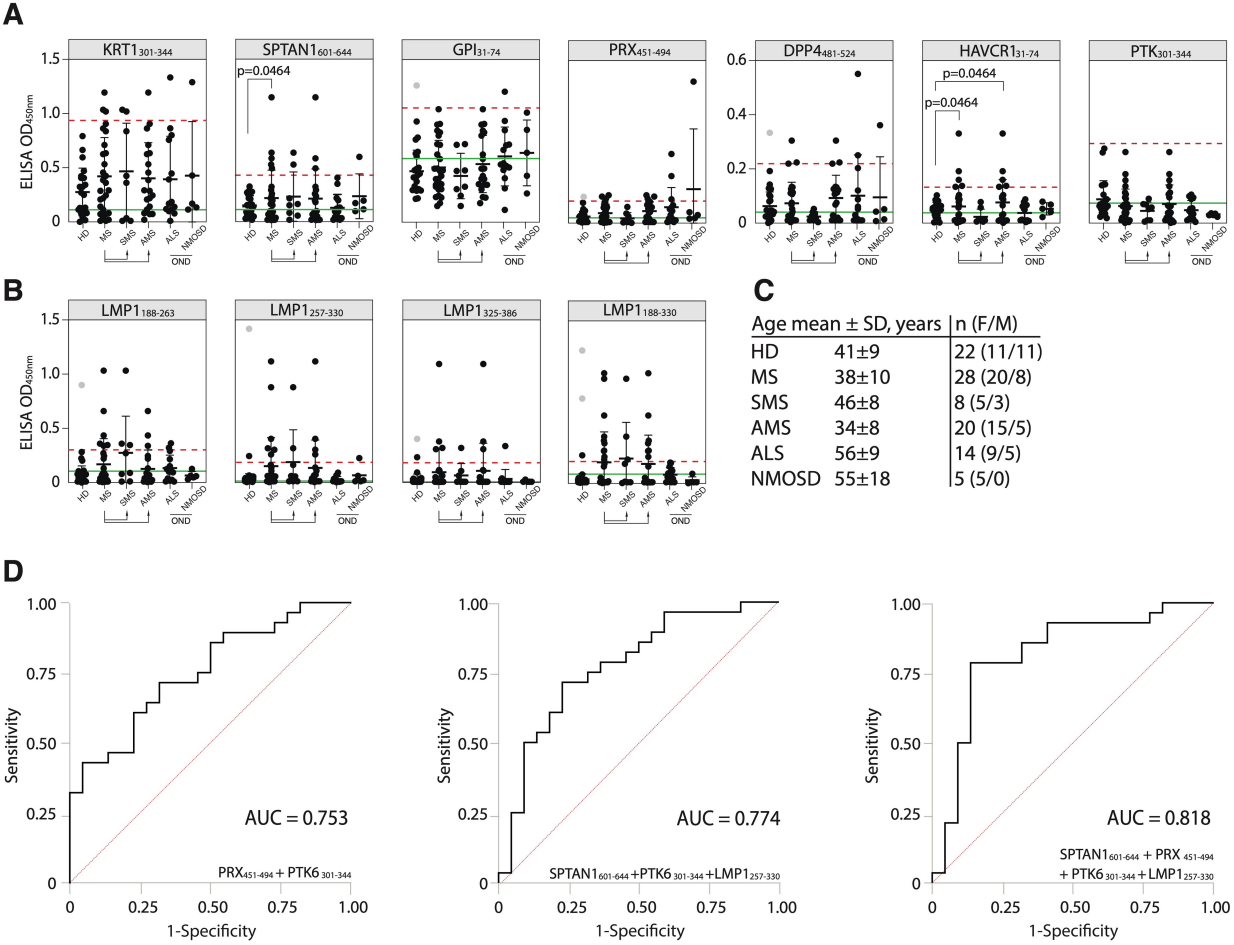
Validation of the discovered antigens. **(A)** ELISA validation of binding activity of serum antibodies from MS, ALS, NMOSD patients and HD with discovered auto-peptides. **(B)** Binding of serum antibodies from MS, ALS, NMOSD patients and HD with the EBV LMP1 C-terminal fragments 188-263, 257-330, 325-386, 188-330 a.a. measured by ELISA and displayed as dot diagrams. Each dot represents individual donor. Mean values ± SD (error bars) are shown. Dashed red line indicates the mean of healthy control signal + 3 standard deviations. Outliers within the HD group that were excluded from cutoff calculation are shown in gray. Green line – negative control representing binding of IVIG (pooled IgG from 1000 healthy donors) for each peptide. The Fisher’s exact test was used to determine the statistical significance of the differences in the number of hits that exceeded the three standard deviation cutoffs between donor groups. Only p-values less than 0.05 are indicated to show statistically significant differences. **(C)** Age and gender comparison of MS, ALS, NMOSD and HD analyzed in the same validation experiment. **(D)** ROC-curves of multiple sclerosis patients and healthy controls, according to the titers of serum IgG against designated antigens. ROC curves were constructed using the optimal combination of two or three autoantigens, both with and without the LMP1 fragment.

To determine the suitability of the identified antigens as potential markers for MS monitoring, an additional validation was conducted. New independent HD and patients with MS, amyotrophic lateral sclerosis (ALS), and neuromyelitis optica spectrum disorders (NMOSD) were included (for clinical data, refer to **Supplementary Table S2**) to assess the performance and specificity of the best candidate autoantigens across these diverse neurological conditions. In the new cohort, the number of MS patients with elevated titers of autoantibodies against the SPTAN1_601-644_ peptide was significantly higher than in the HD group (**Figure 6A**). The reproducible and statistically significant difference in the number of SPTAN1 seropositive patients in both the initial and validation cohorts provides strong evidence for the potential utility of anti-SPTAN1 IgG as a diagnostic marker for MS. Despite the high heterogeneity of MS, no significant association was observed between IgG serum binding to antigenic peptides, EDSS score or disease duration (**Figures 6B, C**; **Supplementary Figures S8**–**S10**).

To further assess the diagnostic accuracy of the identified antigens, ROC analysis was also performed (**Figure 6D**, **Supplementary Table S6**). We evaluated whether combinations of several biomarkers selected by combinatorial analysis (45) would result in high predictive power. PTK6_301-344_ and PRX_451-494_ combination demonstrated the highest sensitivity (SE) and AUC value for two-peptide panel (**Figure 6D**; **Supplementary Table S6**). The antibodies to viral protein LMP1_257-330_ fragment can be used as potential biomarker for MS as well (**Figure 6D**). Employing a triple and four-biomarker panel for simultaneous binding of an autoantigens and viral LMP1 allowed improving the predictive values of ROC curves (**Figure 6D**). The simultaneous use of four biomarkers (SPTAN1_601-644_, PRX_451-494_, PTK6_301-344_ and LMP1_257-330_) showed the highest prediction value for stratifying subjects with MS (n = 28) from healthy controls (n = 22) providing balanced sensitivity of 79% and specificity of 86% (AUC = 0.818) (**Figure 6D**). To estimate the overall biomarker performance, a permutation test was carried out by resampling the observed data (**Supplementary Figure S11**). Obtained results imply that analyzed marker combinations provide robust sample classification.

### 3.6 Validation of elevated IgG levels against identified antigens in the CSF of patients with MS

**Figure 7:**
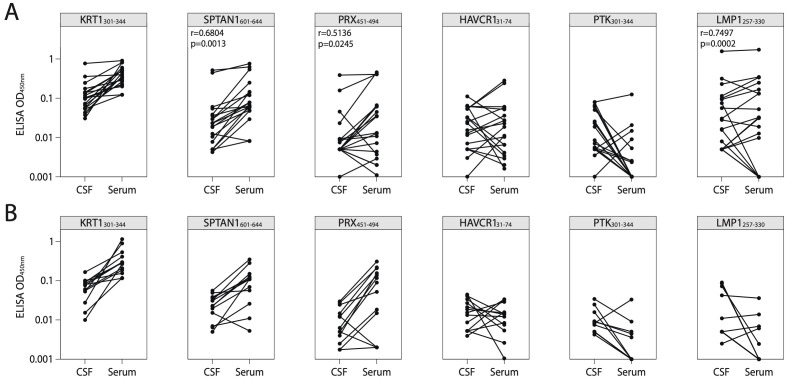
Correlation of antigen-specific IgG levels between serum and CSF. ELISA analysis of IgG binding activity with discovered peptide antigens measured in paired CSF and serum samples of patients with **(A)** MS or **(B)** ALS. Each dot represents individual donor (n=19 for MS; n=13 for ALS). All associations were tested with the Pearson correlation coefficient. Only p-values less than 0.05 are indicated to show statistically significant differences. ALS, amyotrophic lateral sclerosis; MS, multiple sclerosis; CSF, cerebrospinal fluid.

To confirm the presence of autoreactive antibodies targeting the identified peptides in CNS during MS, we examined paired CSF and serum samples from patients with SMS, AMS, and ALS (for clinical data, refer to **Supplementary Table S3**). Firstly, CSF NfL, a marker of axonal injury, was measured. Notably, in line with previous studies, CSF NfL levels correlated with disease activity (47, 48) and were significantly higher in AMS compared to SMS (**Supplementary Figure S12A**). Simultaneously, patients with ALS exhibited elevated CSF NfL levels when compared to both SMS and AMS supporting previous research findings (49). Increased titers of CSF autoreactive antibodies targeting the most reliable discovered autoantigen SPTAN1_601-644_ were observed in two out of nine analyzed patients with AMS, while none of the ten patients with SMS or thirteen patients with ALS exhibited similar increased titer (**Supplementary Figure S13A**). Moreover, in patients with MS, the antibody titers of anti-SPTAN1_601-644_, anti-PRX_451-494_, and anti-LMP1_257-330_ IgG in CSF positively correlated with the levels of serum IgG which target the same antigens (**Figure 7**). In contrast, CSF from ALS patients showed no immunoreactivity to any of the six tested antigens (**Supplementary Figure S13**), suggesting that the response is specific to MS. Patients with AMS had higher IgG concentrations in CSF than patients with SMS and ALS (**Supplementary Figure S12B**). Both patients with elevated titers of anti-SPTAN1_601-644_ IgG in CSF (**Supplementary Figure S13**) also demonstrated increased total IgG levels in the CSF (**Supplementary Figure S12**). Given that ALS patients exhibited significantly elevated NfL levels compared to MS patients, we assume that the autoantibodies targeting the identified autoantigens observed in MS group are unlikely a result of non-specific axonal damage and neuroinflammation.

## 4 Discussion

PhIP-Seq is a versatile tool used to screen libraries of the complete human peptidome (50), viral peptides [VirScan (51)], microbial and environmental toxins [ToxScan (52)], microbiomes (53), and allergenic peptides [AllerScan library (54)]. Customized phage libraries enable high-resolution profiling of the antibody responses to pathogens (55) and autoantigens (56). Large-scale PhIP-Seq screening of patients with different autoimmune disorders implied that the majority of autoreactive responses were unique among patients with MS (25). Furthermore, recent studies have revealed that only approximately 10% of patients with MS share an autoantibody signature against a common motif (57). The PhIP-Seq screening results presented here reveal the remarkable poly- and cross-specificity of antibodies against self-peptides in MS patients. Along with applying the coding libraries to non-human proteomes, the libraries can be targeted to specific self-proteins that are likely to contain pathogenic epitopes. In this study, we focused on the proteins presumably involved in autoimmune pathologies and created a DNA-encoded bacteriophage library representing autoimmune-associated proteins. We replaced T7 bacteriophage with fd bacteriophage and employed the PhIP-Seq technology to identify novel autoimmune targets in the serum of MS patients.

Using the PhIP-Seq immunoprofiling, we observed diverse IgG responses among MS patients. Despite this fact, several autoantigens were common for a portion of MS patients. Validating of candidate peptides required producing them in bacteria and testing of their reactivity with serum IgG by ELISA and SPR. Although immunoreactivity to various single antigens in peripheral blood and CSF of MS patients have been reported earlier (22, 25, 58), our data extend and expand these findings, highlighting a heterogeneous antibody response between MS patients, and allowed us identifying new antigens with potential of being reliable biomarkers. Furthermore, we discovered a set of antibodies to MS-associated peptides and a viral peptide that enable distinguishing MS patients and healthy donors. To the best of our knowledge, the novel autoantigens identified in this work have not been previously reported as MS-related. The diagnostic potential of autoantibodies to SPTAN1, a membrane scaffold protein abundant in neurons of CNS and peripheral nervous system (PNS) was assessed (59). Being a structural protein, SPTAN1 is located in the inner space of axons and normally is not exposed to the immune system. Here we also report autoantibodies to PRX, neuronal protein from the PNS, that is required for normal remyelination after nerve injury. Antibodies to PRX were previously found in the sera of patients with diabetes mellitus and monoclonal gammopathy of undetermined significance (60). Thus, autoantibodies against SPTAN1 and PRX products may indicate nerve tissue loss in the CNS and the PNS. Our study also identified autoantibodies to PTK6 in the sera of MS patients. While PTK6 is mainly associated with cancer (61), it is also expressed in EBV-transformed B cells, suggesting a possible indirect link between this antigen and MS disease. Despite these findings, significant questions regarding the origin, role, and heterogeneity of the autoimmune response to these antigenic epitopes in the normal physiology and pathology of the nervous system remain unresolved. We found that different types of MS (SMS and AMS) are associated with cross-reactivity of autoreactive antibodies (**Figure 5B**).

Recently, one of the MS triggers is believed to be the molecular mimicry between the EBV protein EBNA1 and human CNS autoantigens: Glial-CAM (12, 62) or CRYAB (22) for B cells and MBP for T cells (63). Since only around 20% of MS patients have the EBNA1 – GlialCAM or EBNA-1 – CRYAB cross-reactive antibodies, other cross-reactive antigens remain to be identified. Viral proteins associated with MS are likely: (*i*) to belong to an ubiquitous and highly seropositive virus; (*ii*) to be expressed during the latent phase allowing for the virus-host coexistence; and (*iii*) to encode proline rich (64), glycine-rich (65), or some other common motifs implicated in MS. To date, researchers have focused on studying the immunogenicity of EBNA1 and its role in MS progression, while neglecting some other EBV proteins. Here we investigated the association of MS with other EBV latency-associated protein LMP1, required along with EBNA1 for efficient B cell immortalization. The existing data (19, 23) imply that along with cross-reactivity between human autoantigens and EBNA1, MS can be associated with cross-reactivity between human autoantigens and LMP1. Detailed analysis of the expression of EBNA1, LMP1 and other EBV proteins during latent or lytic infection can reveal their correlation with MS progression.

**Figure 8:**
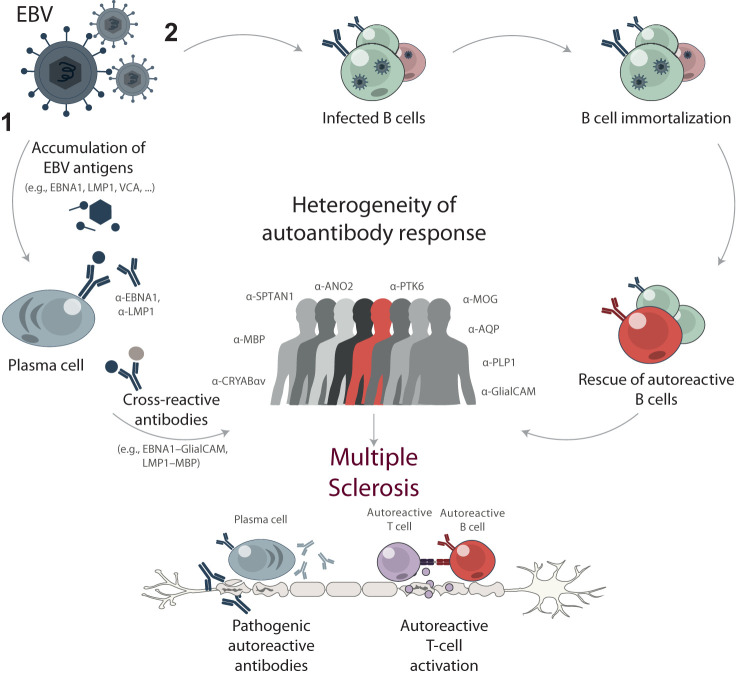
Proposed mechanisms of EBV contribution to multiple sclerosis development through B cell response. High EBV viral load and long-term exposure are proposed to trigger or drive MS development through two main mechanisms: (1) cross-reactivity and molecular mimicry, and (2) reprogramming and immortalization of pathogenic autoreactive B cells. The mechanism of cross-reactivity and molecular mimicry involves the accumulation of EBV antigens, such as EBNA1, LMP1, and VCA, resulting in elevated titers of anti-EBV antibodies. Some of these antibodies cross-react with self-antigens in the central nervous system, contributing to the progression of MS. Alternatively, EBV acts as a chronic source of immortalized B cells with various specificities, including autoreactive ones. These autoreactive B cells can contribute to the pathogenesis of MS by producing self-reactive antibodies and presenting autoantigens to autoreactive T cells. The stochastic nature of immune response development during both mechanisms results in a high degree of heterogeneity in autoreactive antibodies among MS patients.

Cross-reactivity and molecular mimicry are not the only mechanisms underlying the impact of EBV on MS development. Another type of viral effects is B cells reprogramming (**Figure 8**). EBV immortalization of autoreactive B cell clones was proposed more than 10 years ago as a possible MS trigger (66). Thereby, EBV transformation allows the normal elimination of autoreactive B cells to be bypassed. Recently, it was shown that the immortalized human B cells from MS patients might fail controlling EBV latency, and deviated lytic activation promotes inflammatory phenotype of B cells (67). Moreover, the long-term monitoring of EBV-infected individuals revealed that the kinetics of the viral load greatly varies in human population, some of the infected B cells displaying a more rapid expansion than others (68). This explains the elevated levels of autoreactive B cells and the presence of antibodies with different specificities and is in line with the high heterogeneity of the immune response during MS.

Evaluating serum IgG levels against the combination of the identified novel autoantigens and viral LMP1 fosters further research into the development of autoimmune inflammation. The obtained data imply that the identified proteins including SPTAN1_601-644_, PRX_451-494_, PTK6_301-344_ and LMP1 epitopes will be likely used for MS diagnostics. The biomarker risk score model established the novel antigens as blood-based biomarkers of MS with sensitivity [SE] = 79% and specificity [SP] = 86%. CSF analysis of a minor cohort revealed elevated IgG titers specific to SPTAN1_601-644_, PRX_451-494_, and LMP1, but not PTK6_301-344_, exclusively in patients with AMS. CSF antigen-specific IgG levels positively correlate with serum antigen-specific IgG levels and concentration of total IgG in the CSF of patients with MS.

This study has several limitations. The small number of patients with elevated antigen-specific levels in CSF restricts our ability to draw effective correlations with disease progression, NfL levels, or other potential associations. In order to use the proposed panel of antigens as prognostic biomarkers of MS, it is necessary to set up a prospective study of hundreds of patients years before and subsequently after the MS onset. We expect that further longitudinal studies including extensive CSF analysis would support our findings and help establishing the role of these antigens in MS pathogenesis. Recent observations suggest that the serum neurofilament light chain (sNfL) can serve as a biomarker at the very early stages of MS (69) and the combination of NfL with other potential biomarkers in serum or CSF showed high MS prediction score (70). The diagnostic value of the discovered proteins requires further research, and we hope that the identified peptides combined with sNfL concentration analysis would become a convenient, cost effective and reliable serologic biomarker for MS diagnostics and prognosis.

## Data Availability

The datasets presented in this study can be found in online repositories. The names of the repository/repositories and accession number(s) can be found below: NCBI SRA repository PRJNA1070874.
