# Supplementary material for: High heterogeneity of cross-reactive immunoglobulins in multiple sclerosis presumes combining of B-cell epitopes for diagnostics: a case-control study
Source: Front Immunol. 2024 Nov 28;15:1401156. doi: 10.3389/fimmu.2024.1401156 (PMC11634884; doi:10.3389/fimmu.2024.1401156)
Supplement: Supplementary file 1 [file DataSheet1.docx]

**Supplemental Material**

**High heterogeneity of cross-reactive immunoglobulins in multiple sclerosis presumes combining of B cell epitopes for diagnostics: a case-control study**

**by Ovchinnikova et al.**

**Figure S1. Representation of the autoantigen library.** 94% of all library clones are detected after sequencing to a depth of 32x.


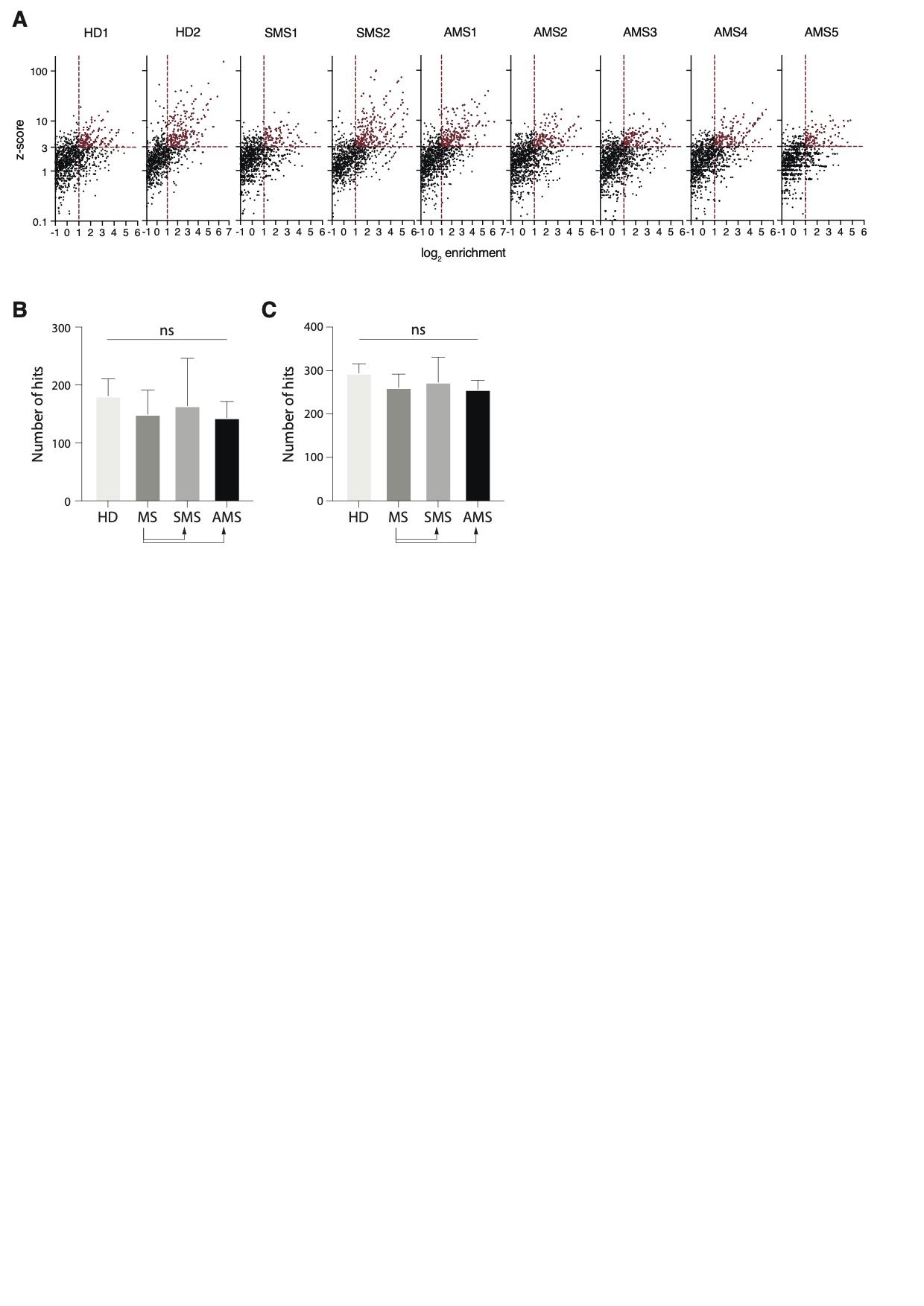


**Figure S2. Bioinformatic analysis of human peptidome library enrichment against serum IgG.** PhIP-seq captures a similar number of seroactive peptides in MS patients and healthy donors (**A**, **B**, **C**). (**A**) The scatter plots illustrate the enrichment analysis against serum IgG from healthy donors (HD1, HD2), patients with stable multiple sclerosis (SMS1, SMS2) or active multiple sclerosis (AMS1 – AMS5) according to z-score and enrichment filtration. Each dot represents a distinct peptide. Peptides colored in red are considered significantly enriched for the analyzed donor. The breadth of antibody reactivity, shown as the number of seroactive peptides in MS patients and HD (number of enriched peptides), according to (**B**) z-score and enrichment criteria or (**C**) z-score only. The data shown are mean ± SD.


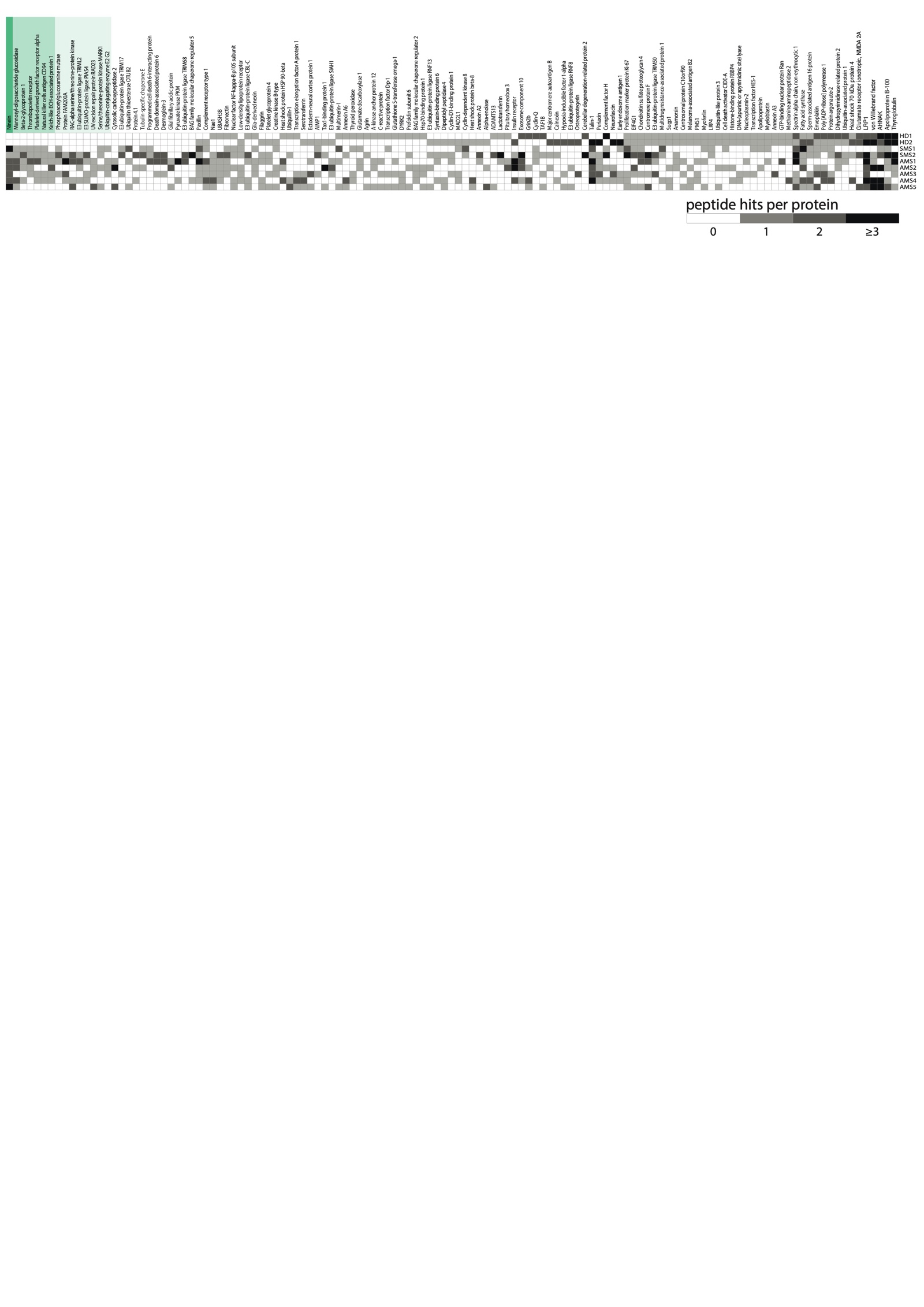


**Figure S3. Autoantigen proteins identified by PhIP-Seq.** A heatmap showing proteins identified as seroactive and show specificity to healthy donors and/or MS patients. Peptide hits per protein indicate the number of peptides from indicated protein. Each row represents one individual. Proteins are sorted in ascending order of the IgG response in healthy donors. SMS, stable multiple sclerosis; AMS, active multiple sclerosis.


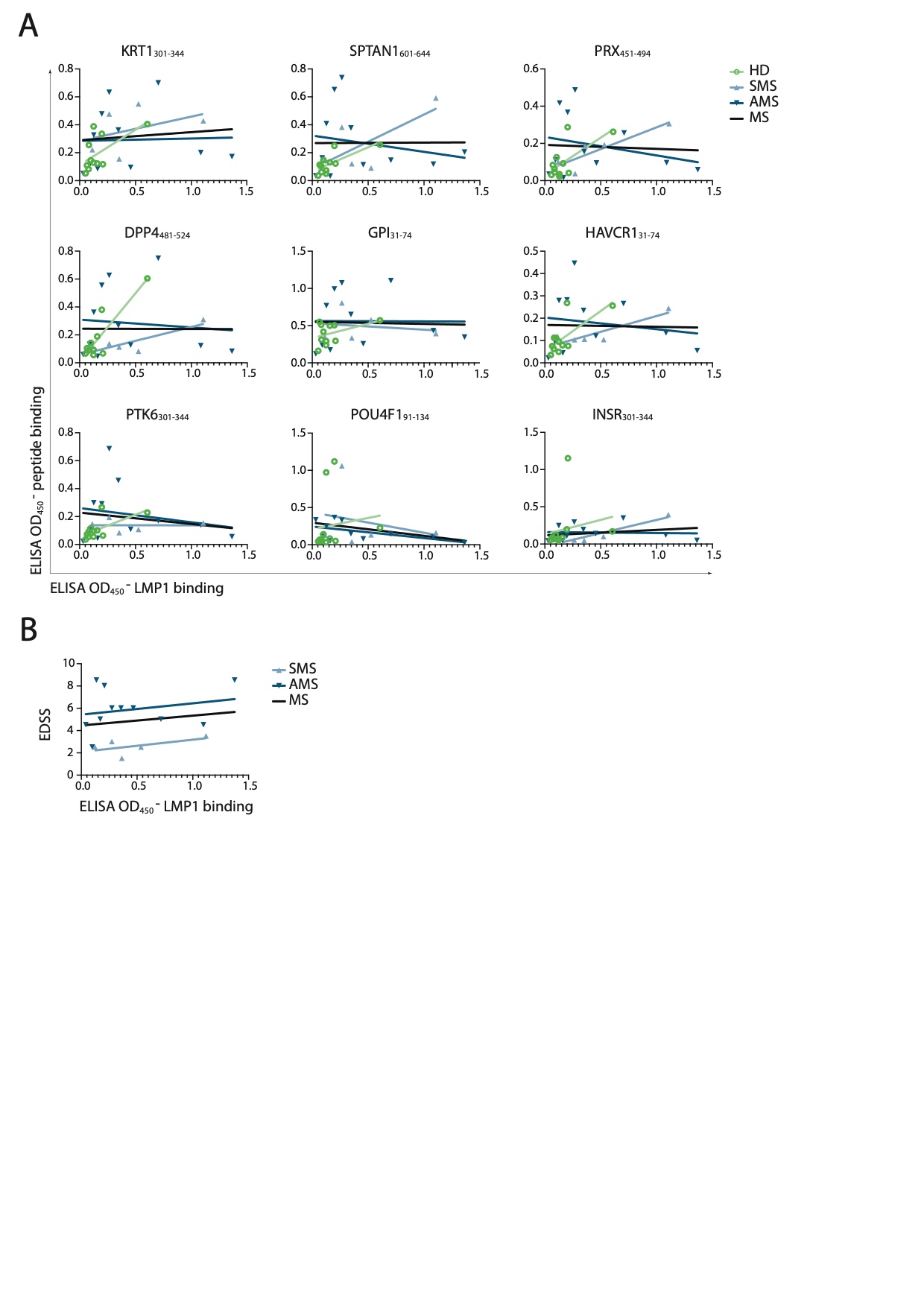


**Figure S4. Correlation of IgG binding to LMP1 and to human autoantigens.** (**A**) No statistically significant correlation between the level of antibodies recognizing LMP1 and analyzed autoantigens was detected in serum from HD or MS. (**B**) Lack of significant correlation between the MS severity (according to EDSS score - Expanded Disability Status Scale) and the titer of anti-LMP1 antibodies in MS patients (SMS and AMS). The binding of serum IgG to full-length LMP1 and identified potential autoantigens was measured by ELISA. Trend lines are colored in green for HD, black for MS, light blue for SMS, and sapphire for AMS.


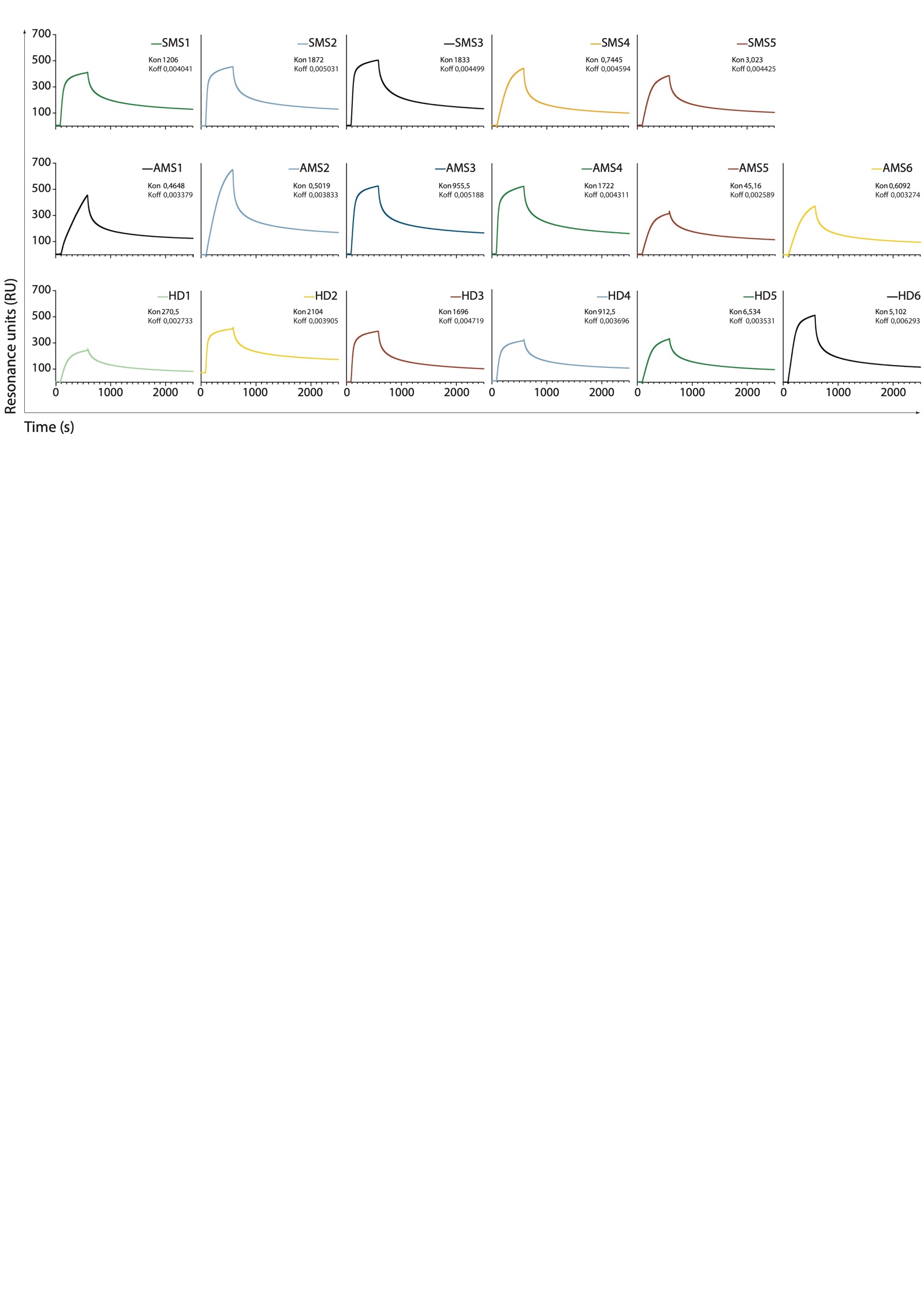


**Figure S5. Biacore binding curves.** Kinetic analysis of SPTAN1_601-644_ binding by IgG from MS patients’ and HD serum. To eliminate the influence of serum antibody non-specific binding with the TRX-carrier, we analyzed the interaction with empty TRX as a reference and subtracted its signal from acquired SPTAN1_601-644_-TRX data. RU: resonance units. SMS, stable multiple sclerosis; AMS, active multiple sclerosis.

**
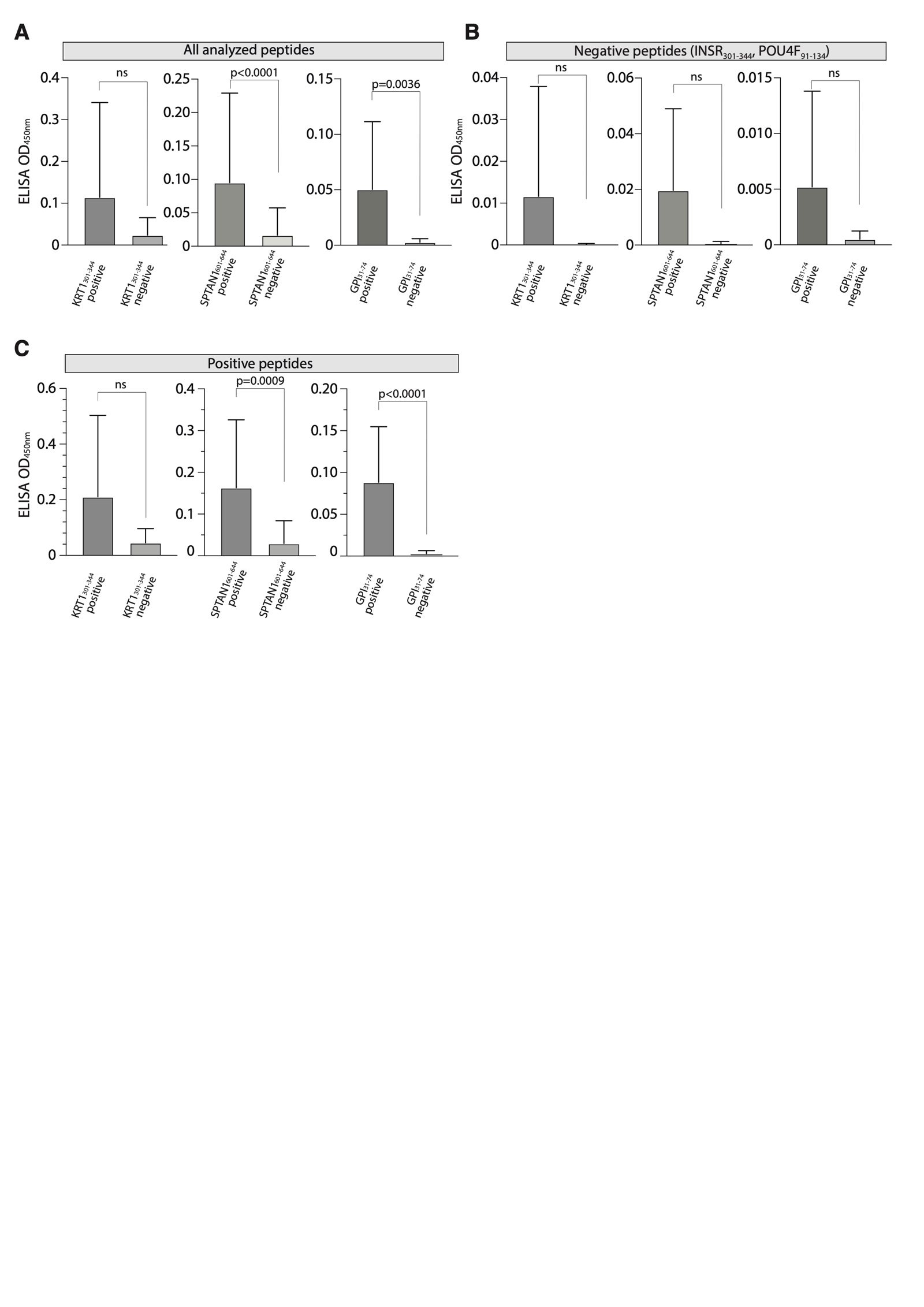
**

**Figure S6. Isolated autoantibodies from the serum of MS patients reveal elevated cross-reactivity with human PhIP-identified autoantigens.** The binding activity of antigen-enriched positive and negative IgG against (**A**) all analyzed autoantigen peptides (KRT1_301-344_, SPTAN1_601-644_, DPP4_481-524_, PRX_451-494_, HAVCR1_31-74_, PTK6_301-344_, GPI_31-74_, INSR_301-344_ and POU4F1_91-134_), (**B**) autoantigen peptides with no detected immunogenicity during MS (INSR_301-344_ and POU4F1_91-134_), (**C**) autoantigen peptides with the highest detected immunogenicity during MS (KRT1_301-344_, SPTAN1_601-644_, DPP4_481-524_, PRX_451-494_, PTK6_301-344_, GPI_31-74_). Binding activity against enriched peptides is not included in the analysis (**A**, **C**). Data are shown as mean ± SD, and p-values. The statistical significance was evaluated with the Mann Whitney test with a false discovery rate (FDR) correction (only significant p-values are shown).


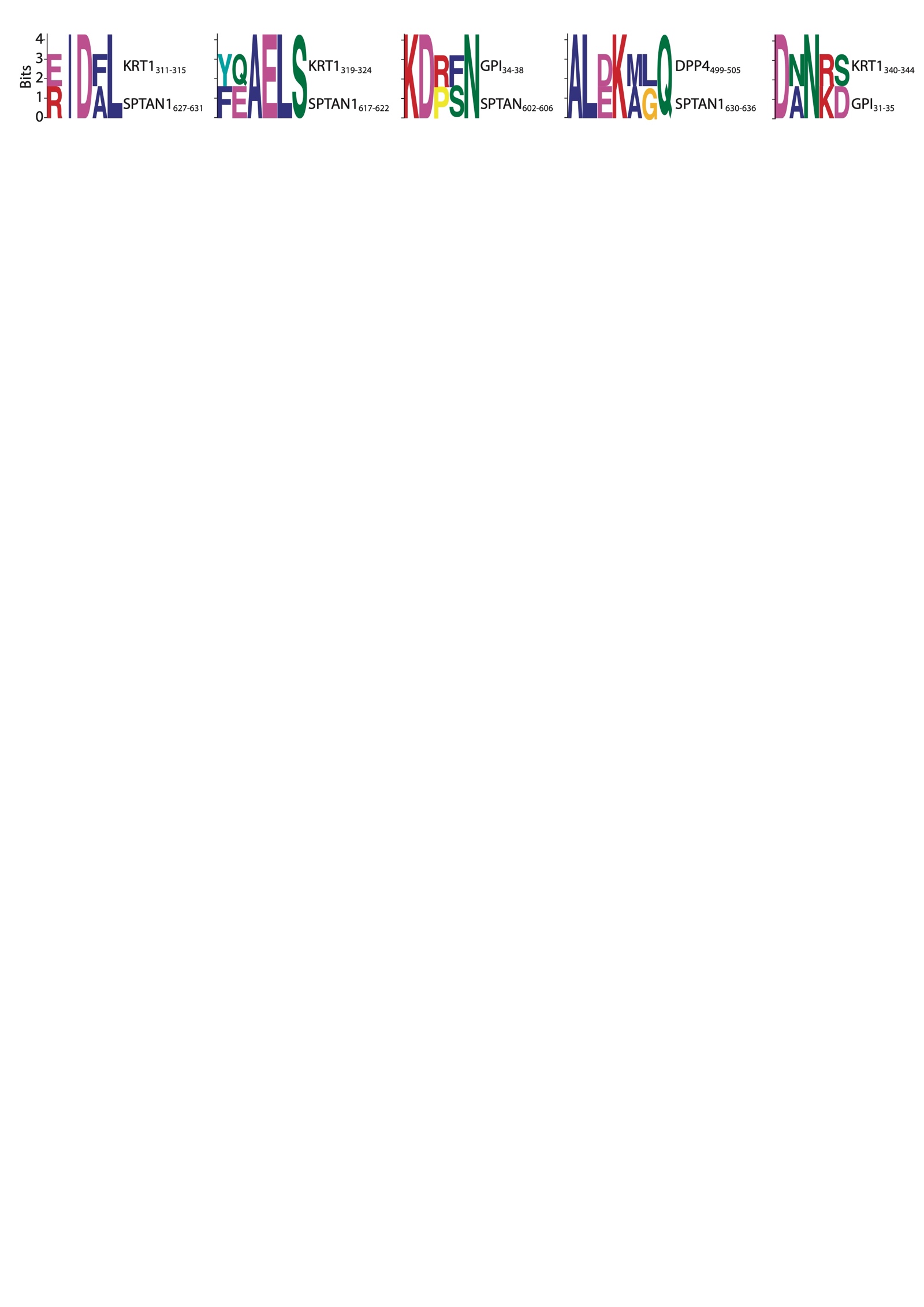


**Figure S7 Comparison of autoantigen amino acid sequences recognized by serum IgGs from MS patients.** Pairwise comparison of identified autopeptides (logos were created with Two-Sample-Logos by MEME algorithm); the height of symbols within the stack in each logo indicates the relative frequency of each amino acid at that position. Amino acids: hydrophobic (blue), polar uncharged side chains (green), with electrically charged side chains (red), proline and glycine (yellow and orange).


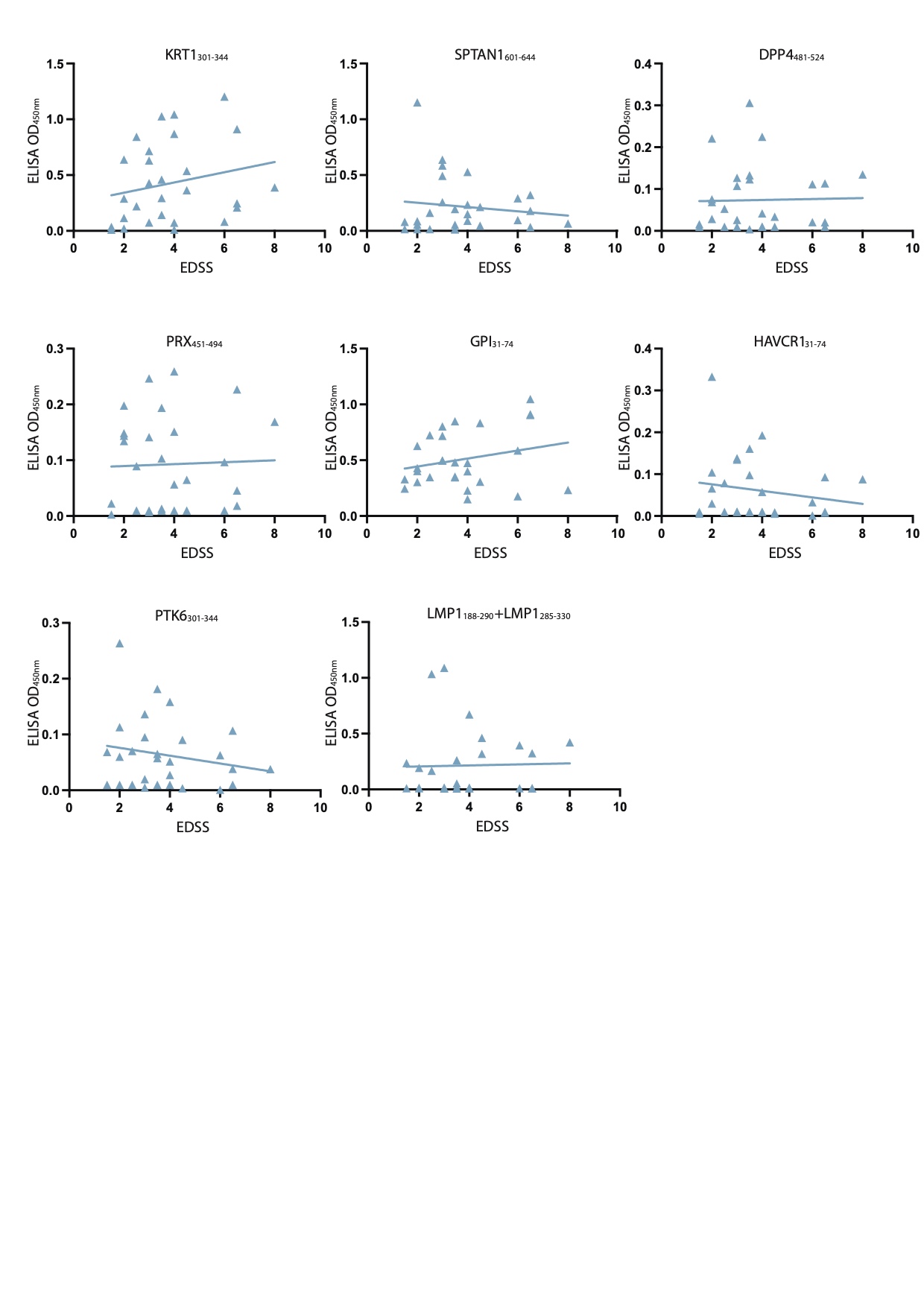


**Figure S8. Correlation of IgG binding with EDSS.** Lack of statistically significant correlation between the MS severity (according to EDSS score) and the titer of the antibodies against analyzed autoantigens in MS patients. The binding of serum IgG with analyzed antigens fused with TRX-carrier was measured by ELISA.


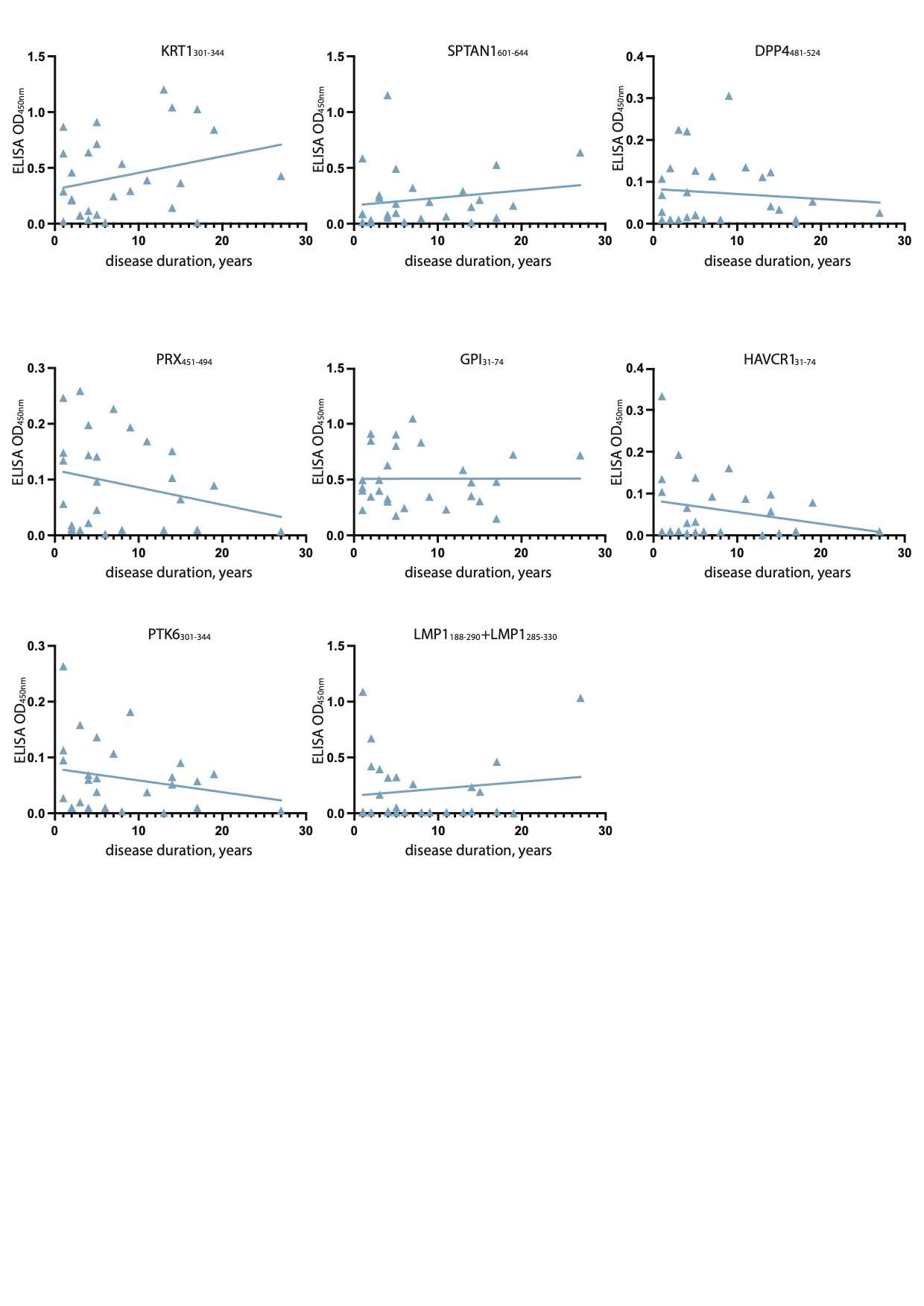


**Figure S9 Correlation of IgG binding with disease duration.** Lack of statistically significant correlation between the disease duration and the titer of the antibodies against analyzed autoantigens in MS. The binding of serum IgG with analyzed antigens fused with TRX-carrier was measured by ELISA.


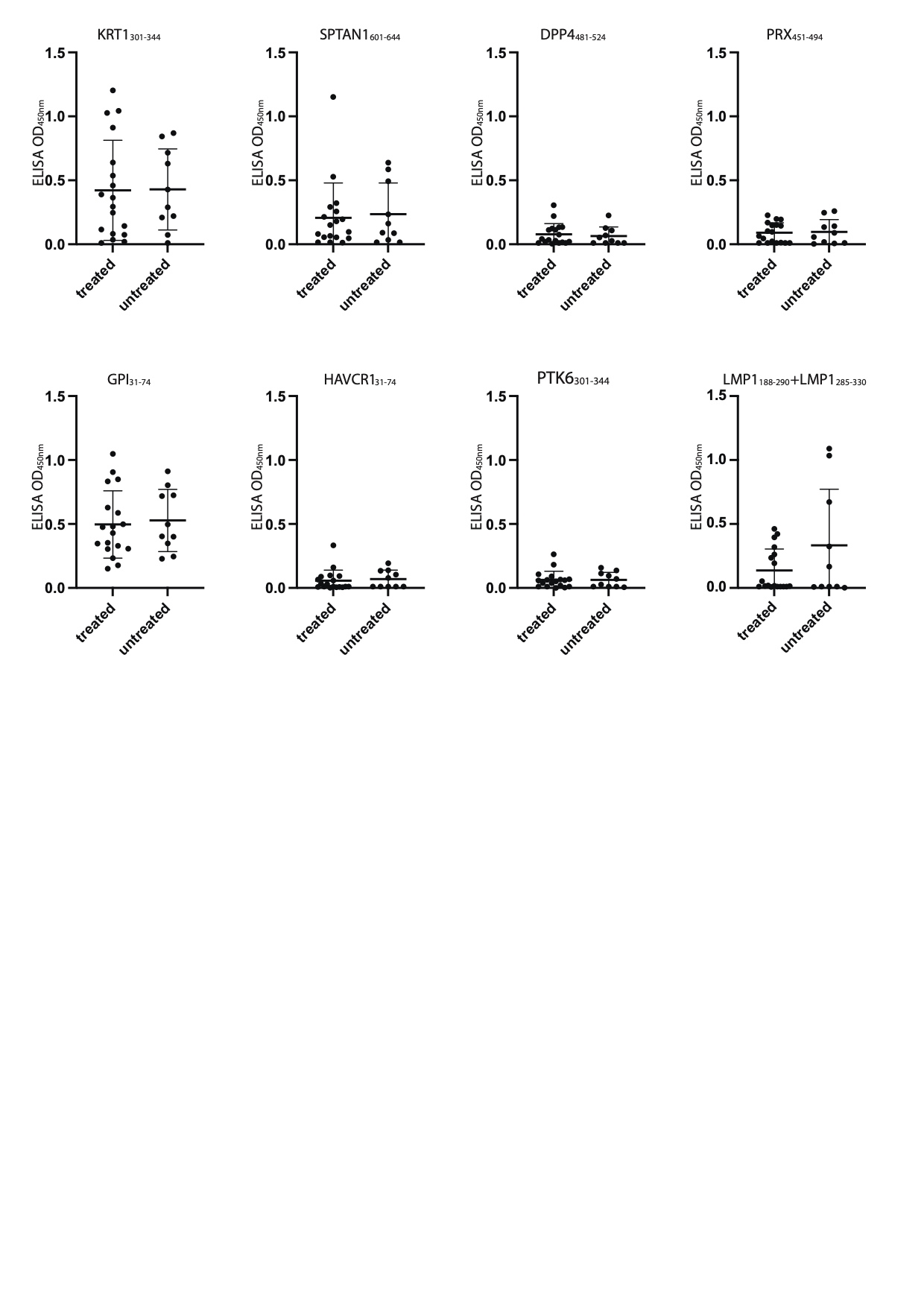


**Figure S10. Correlation of IgG binding with treatment of MS.** Lack of statistically significant dependence of the antibodies’ titers in MS patients and MS treatment history. The binding of serum IgG with analyzed antigens fused with TRX-carrier was measured by ELISA. None of the patients received glucocorticoid treatment or immunomodulatory treatment for at least 6 months prior to blood collection. Donor was considered “treated” if MS treatment was received at least once. The Mann-Whitney test was used to determine statistical significance of values obtained for different donor groups.


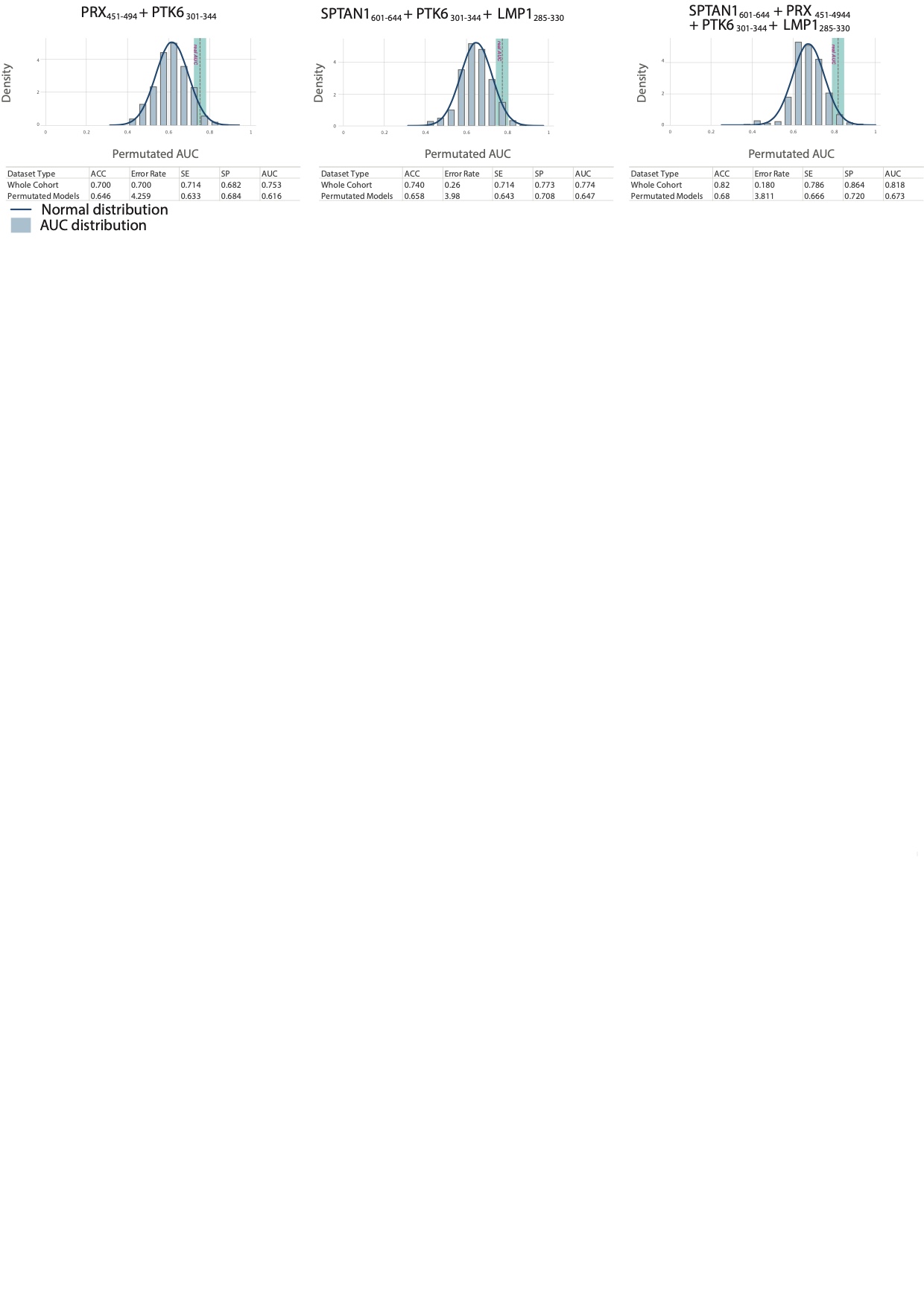


**Figure S11. ROC validation of MS against HD.** Density distribution of permutated AUC values compared to the normal distribution. In the permutated models, the ‘real’ AUC values were found outside the reference density distribution in the MS group, rendering them models with high validity. The ‘real AUC values’ lay outside the permutated AUC distribution that indicate a high validity of the marker panels generated via CombiROC [(1)](https://sciwheel.com/work/citation?ids=7012284&pre=&suf=&sa=0&dbf=0).


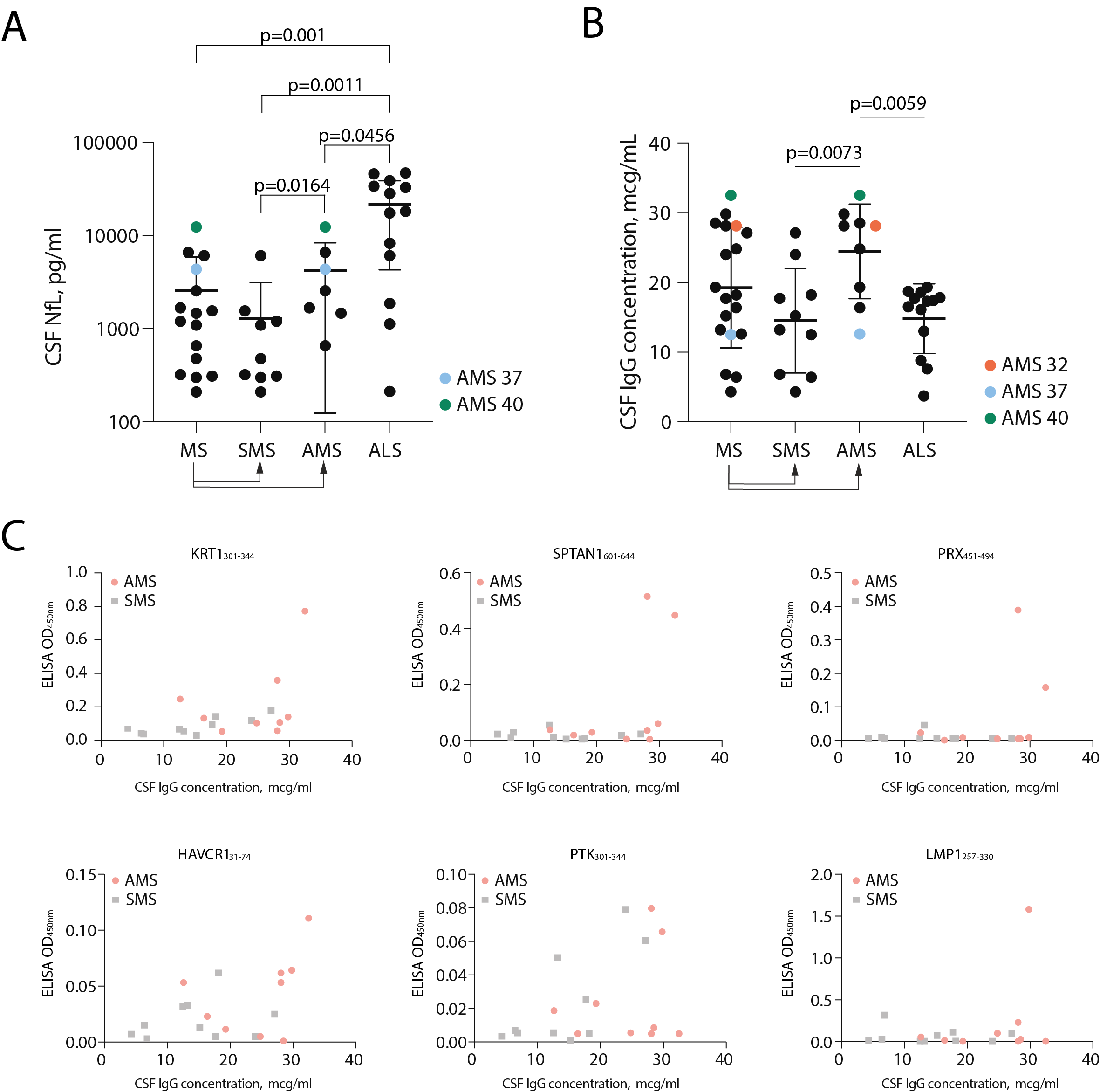


**Figure S12. NfL and IgG levels in CSF of patients with MS and ALS.** (**A**) CSF NfL concentrations across patients with different courses of MS and individuals with ALS. (**B**) CSF IgG concentrations across patients with different courses of MS and individuals with ALS. Individual donors with elevated levels of anti-SPTAN1 IgG in serum are colored. The Mann-Whitney test was used to determine statistical significance of values obtained for different donor groups. Only p-values less than 0.05 are indicated to show statistically significant differences. (**C**) Analysis of the correlation between level of Ag-specific IgG in CSF and total IgG concentration in CSF. ALS – amyotrophic lateral sclerosis; MS – multiple sclerosis; AMS – active MS; SMS – stable MS; CSF – cerebrospinal fluid.


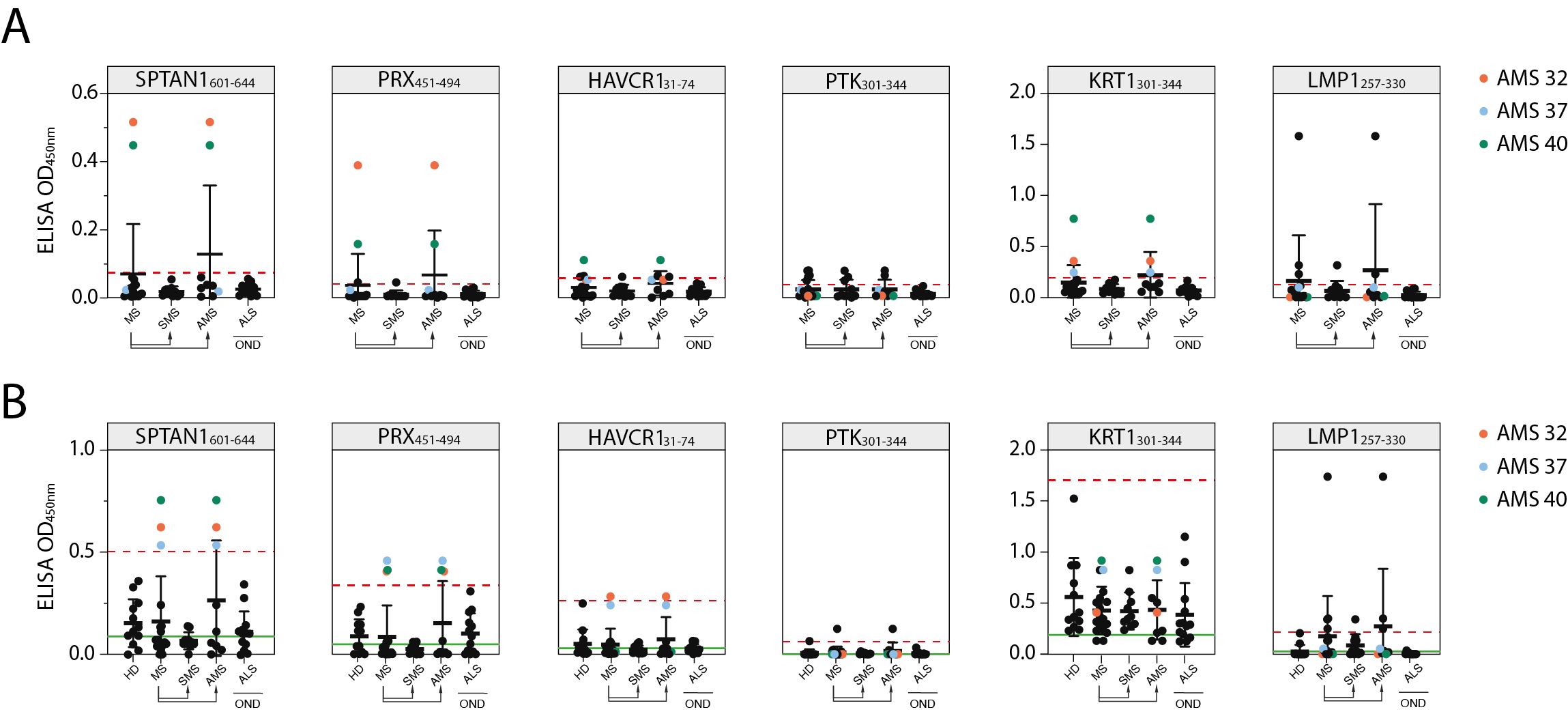


**Figure S13.** **CSF and serum Ag-specific IgG titer measurement.** ELISA validation of binding activity of CSF (**A**) and serum (**B**) antibodies from MS and ALS patients with discovered auto-peptides and viral LMP1 C-terminal _257-330_ fragment. Mean values ± SD (error bars) are shown. Dashed red line indicates the mean of HD and ALS signal + 3 standard deviations for serum and CSF analysis, respectively. Green line ­– negative control representing binding of IVIG (pooled IgG from 1000 healthy donors) for each peptide. Each dot represents individual donor. Individual donors with elevated levels of anti-SPTAN1 IgG in serum are colored. ALS – amyotrophic lateral sclerosis; MS – multiple sclerosis; AMS – active MS; SMS – stable MS; CSF – cerebrospinal fluid; OND – other neurological diseases.

**Table S1. Baseline and clinical characteristics of patients with relapsing-remitting multiple sclerosis and healthy donors in the autoantigen screening phase. Cohort 1.**

| # | Donor | Age, years | Sex | EDSS | Therapy before study, years | Disease duration, years | PhIP-Seq analysis | ELISA testing |
| --- | --- | --- | --- | --- | --- | --- | --- | --- |
| 1 | AMS1 | 33 | Male | 6 | IFNβ1b (1-2; 8-12) | 12 | + | + |
| 2 | AMS2 | 32 | Male | 6 | IFNβ1b (1*; 9-11) | 11 | + | + |
| 3 | AMS3 | 39 | Female | 8.5 | No treatment | 3 | + | + |
| 4 | AMS4 | 29 | Female | 8 | GA (3-5), IVIG (3), IFNβ1b (1-2) | 12 | + | + |
| 5 | AMS5 | 22 | Female | 4.5 | No treatment | 1 | + | + |
| 6 | AMS6 | 23 | Male | 5 | No treatment | 3 | - | + |
| 7 | AMS7 | 37 | Female | 5 | IFNβ1b (2-4), GA (1-2) | 5 | - | + |
| 8 | AMS8 | 46 | Male | 6 | IFNβ1b (1) | 2 | - | + |
| 9 | AMS9 | 44 | Female | 4.5 | No treatment | 2 | - | + |
| 10 | AMS10 | 24 | Male | 2.5 | No treatment | 2 | - | + |
| 11 | AMS11 | 44 | Male | 8.5 | IFNβ1b (2-3) | 13 | - | + |
| 12 | SMS1 | 43 | Female | 1.5 | No treatment | 12 | + | + |
| 13 | SMS2 | 36 | Male | 2.5 | No treatment | 14 | + | + |
| 14 | SMS3 | 58 | Female | 3.5 | No treatment | 30 | - | + |
| 15 | SMS4 | 56 | Female | 2.5 | No treatment | 11 | - | + |
| 16 | SMS5 | 61 | Female | 3 | No treatment | 26 | - | + |
| 17 | HD1 | 27 | Female | N/A | N/A | N/A | + | + |
| 18 | HD2 | 22 | Female | N/A | N/A | N/A | + | + |
| 19 | HD3 | 25 | Female | N/A | N/A | N/A | - | + |
| 20 | HD4 | 42 | Female | N/A | N/A | N/A | - | + |
| 21 | HD5 | 24 | Female | N/A | N/A | N/A | - | + |
| 22 | HD6 | 47 | Male | N/A | N/A | N/A | - | + |
| 23 | HD7 | 51 | Female | N/A | N/A | N/A | - | + |
| 24 | HD8 | 40 | Female | N/A | N/A | N/A | - | + |
| 25 | HD9 | 24 | Female | N/A | N/A | N/A | - | + |
| 26 | HD10 | 35 | Male | N/A | N/A | N/A | - | + |
| 27 | HD11 | 36 | Male | N/A | N/A | N/A | - | + |

MS – multiple sclerosis; AMS – active MS; SMS – stable MS; HD – healthy donors; IFNβ1b – interferon-β-1b; GA – glatiramer acetate; IVIG – intravenous immunoglobulin; EDSS – expanded disability status scale; N/A – not applicable. * None of the patients received glucocorticoid treatment or immunomodulatory treatment for at least 6 months prior to blood collection.

**Table S2. Baseline and clinical characteristics of patients with relapsing-remitting multiple sclerosis, amyotrophic lateral sclerosis, neuromyelitis optica spectrum disorders, and healthy donors, in the validation phase. Cohort 2.**

| # | Group | Age, years | Sex | EDSS | Therapy before study, years | Disease duration, years |
| --- | --- | --- | --- | --- | --- | --- |
| 1 | AMS12 | 39 | Female | 2 | GCs, TPE (1*) | 1 |
| 2 | AMS13 | 23 | Female | 2 | No treatment | 1 |
| 3 | AMS14 | 30 | Female | 2 | GCs (1*) | 4 |
| 4 | AMS15 | 27 | Male | 3.5 | IFNβ1a (1-2) | 2 |
| 5 | AMS16 | 38 | Male | 8 | IFNβ1a (4-9), GA (9-10), Ocrelizumab (1-2) | 11 |
| 6 | AMS17 | 38 | Male | 6.5 | IFNβ1b (1-4) | 7 |
| 7 | AMS18 | 40 | Female | 3.5 | IFNβ1a (1-5) | 9 |
| 8 | AMS19 | 32 | Female | 6 | Fingolimod (9-10), Natalizumab (4-6; 7-8), Alemtuzumab (2; 3) | 13 |
| 9 | AMS20 | 36 | Male | 3 | No treatment | 5 |
| 10 | AMS21 | 23 | Female | 2 | IFNβ1b (1-2) | 4 |
| 11 | AMS22 | 46 | Female | 4.5 | PEG-IFNβ1a (1-2) | 8 |
| 12 | AMS23 | 32 | Female | 6.5 | No treatment | 2 |
| 13 | AMS24 | 28 | Female | 6.5 | IFNβ1b (1-2) | 5 |
| 14 | AMS25 | 34 | Female | 3.5 | GA (1-10) | 14 |
| 15 | AMS26 | 36 | Female | 3.0 | No treatment | 1 |
| 16 | AMS27 | 32 | Female | 3.0 | IFNβ1b (1-2) | 3 |
| 17 | AMS28 | 51 | Female | 4.0 | No treatment | 3 |
| 18 | AMS29 | 48 | Female | 2.5 | No treatment | 2 |
| 19 | AMS30 | 30 | Male | 6.0 | IFNβ1b (3) | 5 |
| 20 | AMS31 | 20 | Female | 4.0 | No treatment | 1 |
| 21 | SMS6 | 33 | Male | 1.5 | No treatment | 6 |
| 22 | SMS7 | 40 | Female | 3.5 | GCs (1*) | 17 |
| 23 | SMS8 | 52 | Male | 4 | GCs (1*) | 14 |
| 24 | SMS9 | 41 | Female | 1.5 | GCs (1*) | 10 |
| 25 | SMS10 | 56 | Female | 3 | No treatment | 27 |
| 26 | SMS11 | 45 | Male | 4 | GA (9-14) | 17 |
| 27 | SMS12 | 43 | Female | 2.5 | No treatment | 19 |
| 28 | SMS13 | 57 | Female | 4.5 | Teriflunomide (1-3) | 15 |
| 29 | ALS1 | 48 | Female | N/A | antioxidant therapy | 2 |
| 30 | ALS2 | 59 | Male | N/A | antioxidant therapy | 2 |
| 31 | ALS3 | 60 | Male | N/A | antioxidant therapy | 4 |
| 32 | ALS4 | 40 | Female | N/A | antioxidant therapy | 4 |
| 33 | ALS5 | 54 | Female | N/A | antioxidant therapy | 2 |
| 34 | ALS6 | 65 | Female | N/A | antioxidant therapy | 2 |
| 35 | ALS7 | 51 | Male | N/A | antioxidant therapy | 2 |
| 36 | ALS8 | 45 | Female | N/A | antioxidant therapy | 4 |
| 37 | ALS9 | 65 | Female | N/A | antioxidant therapy | 1 |
| 38 | ALS10 | 47 | Female | N/A | antioxidant therapy | 1 |
| 39 | ALS11 | 65 | Female | N/A | antioxidant therapy | 2 |
| 40 | ALS12 | 50 | Male | N/A | antioxidant therapy | 1 |
| 41 | ALS13 | 63 | Male | N/A | antioxidant therapy | 2 |
| 42 | ALS14 | 65 | Female | N/A | antioxidant therapy | 1 |
| 43 | NMOSD1 | 70 | Female | N/A | Azathioprine | 18 |
| 44 | NMOSD2 | 46 | Female | N/A | Rituximab | 2 |
| 45 | NMOSD3 | 64 | Female | N/A | Rituximab | 1,5 |
| 46 | NMOSD4 | 29 | Female | N/A | Azathioprine | 9 |
| 47 | NMOSD5 | 68 | Female | N/A | Azathioprine, Rituximab | 5 |
| 48 | HD12 | 41 | Female | N/A | N/A | N/A |
| 49 | HD13 | 35 | Male | N/A | N/A | N/A |
| 50 | HD14 | 45 | Female | N/A | N/A | N/A |
| 51 | HD15 | 22 | Male | N/A | N/A | N/A |
| 52 | HD16 | 42 | Female | N/A | N/A | N/A |
| 53 | HD17 | 40 | Female | N/A | N/A | N/A |
| 54 | HD18 | 39 | Female | N/A | N/A | N/A |
| 55 | HD19 | 38 | Male | N/A | N/A | N/A |
| 56 | HD20 | 29 | Female | N/A | N/A | N/A |
| 57 | HD21 | 53 | Male | N/A | N/A | N/A |
| 58 | HD22 | 37 | Male | N/A | N/A | N/A |
| 59 | HD23 | 34 | Male | N/A | N/A | N/A |
| 60 | HD24 | 41 | Male | N/A | N/A | N/A |
| 61 | HD25 | 40 | Female | N/A | N/A | N/A |
| 62 | HD26 | 37 | Female | N/A | N/A | N/A |
| 63 | HD27 | 33 | Male | N/A | N/A | N/A |
| 64 | HD28 | 35 | Female | N/A | N/A | N/A |
| 65 | HD29 | 45 | Male | N/A | N/A | N/A |
| 66 | HD30 | 58 | Female | N/A | N/A | N/A |
| 67 | HD31 | 60 | Male | N/A | N/A | N/A |
| 68 | HD32 | 55 | Female | N/A | N/A | N/A |
| 69 | HD33 | 33 | Male | N/A | N/A | N/A |

MS – multiple sclerosis; AMS – active MS; SMS – stable MS; HD – healthy donors; ALS – amyotrophic lateral sclerosis; NMOSD – neuromyelitis optica spectrum disorders; GCs – glucocorticoids; TBE – therapeutic plasma exchange; IFNβ1b – interferon-β-1b; IFNβ1a – interferon-β-1a; GA – glatiramer acetate; IVIG – intravenous immunoglobulin; EDSS – expanded disability status scale; N/A – not applicable. * None of the patients received glucocorticoid treatment or immunomodulatory treatment for at least 6 months prior to blood collection.

**Table S3. Baseline and clinical characteristics of patients with relapsing-remitting multiple sclerosis, amyotrophic lateral sclerosis, and healthy donors, in the validation phase. Cohort 3.**

| # | Group | Age, years | Sex | EDSS | Therapy before study, years | Disease duration, years | OCBs pattern |
| --- | --- | --- | --- | --- | --- | --- | --- |
| 1 | AMS32 | 26 | Male | 2.5 | No treatment | 3 | Type 2 |
| 2 | AMS33 | 35 | Female | 4 | No treatment | 5 | Type 1 |
| 3 | AMS34 | 36 | Male | 4 | No treatment | 3 | Type 1 |
| 4 | AMS35 | 28 | Female | 1 | No treatment | 0 | Type 2 |
| 5 | AMS36 | 38 | Male | 2.5 | No treatment | 0 | Type 2 |
| 6 | AMS37 | 65 | Female | 3.5 | No treatment | 4 | Type 2 |
| 7 | AMS38 | 58 | Female | 5.5 | No treatment | 5 | Type 2 |
| 8 | AMS39 | 26 | Female | 2.5 | No treatment | 2 | Type 2 |
| 9 | AMS40 | 48 | Male | 3.5 | No treatment | 2 | Type 2 |
| 10 | SMS14 | 34 | Female | 1.5 | No treatment | 4 | Type 1 |
| 11 | SMS15 | 41 | Male | 2 | No treatment | 16 | Type 1 |
| 12 | SMS16 | 38 | Female | 2 | No treatment | 12 | Type 1 |
| 13 | SMS17 | 28 | Male | 2 | No treatment | 5 | Type 1 |
| 14 | SMS18 | 37 | Female | 5 | No treatment | 13 | Type 1 |
| 15 | SMS19 | 34 | Female | 1 | No treatment | 0 | Type 2 |
| 16 | SMS20 | 36 | Male | 2 | No treatment | 1 | Type 1 |
| 17 | SMS21 | 32 | Female | 2 | No treatment | 0 | Type 2 |
| 18 | SMS22 | 28 | Female | 1.5 | No treatment | 1 | Type 1 |
| 19 | SMS23 | 20 | Male | 1 | No treatment | 0 | Type 2 |
| 20 | ALS15 | 50 | Male | N/A | antioxidant therapy | 1 | N/A |
| 21 | ALS16 | 59 | Male | N/A | antioxidant therapy | 6 | N/A |
| 22 | ALS17 | 53 | Female | N/A | antioxidant therapy | 2 | N/A |
| 23 | ALS18 | 39 | Female | N/A | antioxidant therapy | 1 | N/A |
| 24 | ALS19 | 36 | Female | N/A | antioxidant therapy | 1 | N/A |
| 25 | ALS20 | 63 | Female | N/A | antioxidant therapy | 0 | N/A |
| 26 | ALS21 | 55 | Male | N/A | antioxidant therapy | 1 | N/A |
| 27 | ALS22 | 60 | Male | N/A | antioxidant therapy | 1 | N/A |
| 28 | ALS23 | 56 | Female | N/A | antioxidant therapy | 1 | N/A |
| 29 | ALS24 | 59 | Female | N/A | antioxidant therapy | 1 | N/A |
| 30 | ALS25 | 45 | Female | N/A | antioxidant therapy | 6 | N/A |
| 31 | ALS26 | 41 | Female | N/A | antioxidant therapy | 0 | N/A |
| 32 | ALS27 | 41 | Female | N/A | antioxidant therapy | 0 | N/A |
| 33 | HD34 | 36 | Male | N/A | N/A | N/A | N/A |
| 34 | HD35 | 25 | Male | N/A | N/A | N/A | N/A |
| 35 | HD36 | 29 | Female | N/A | N/A | N/A | N/A |
| 36 | HD37 | 28 | Male | N/A | N/A | N/A | N/A |
| 37 | HD38 | 36 | Female | N/A | N/A | N/A | N/A |
| 38 | HD39 | 54 | Female | N/A | N/A | N/A | N/A |
| 39 | HD40 | 45 | Female | N/A | N/A | N/A | N/A |
| 40 | HD41 | 79 | Female | N/A | N/A | N/A | N/A |
| 41 | HD42 | 41 | Female | N/A | N/A | N/A | N/A |
| 42 | HD43 | 67 | Female | N/A | N/A | N/A | N/A |
| 43 | HD44 | 30 | Female | N/A | N/A | N/A | N/A |
| 44 | HD45 | 30 | Female | N/A | N/A | N/A | N/A |

ALS – amyotrophic lateral sclerosis; MS – multiple sclerosis; AMS – active MS; SMS – stable MS; HD – healthy donors; CSF – cerebrospinal fluid; EDSS – expanded disability status scale; N/A – not applicable; OCBs – oligoclonal bands.

* The type 1 pattern has no OCBs in either the CSF or serum, (absent intrathecal IgG synthesis, normal pattern). The type 2 pattern has CSF-restricted OCBs (intrathecal IgG synthesis), according to Wills et al. [(2)](https://sciwheel.com/work/citation?ids=16719557&pre=&suf=&sa=0&dbf=0). None of the patients received glucocorticoid treatment or immunomodulatory treatment for at least 6 months prior to blood collection.

**Table S5. List of oligonucleotides used to make constructs encoding peptide-TRX fusion proteins.**

| Name | 5’-3’ |
| --- | --- |
| for_INSR | aattggatccgcgccttccaacagatg |
| rev_INSR | aaatccatgggttctggttcctgccatcagtatgtgat |
| for_GPI | aattggatccgcgcttttggccagat |
| rev_GPI | aaatccatgggttctggttccgatgccaacaaagatc |
| for_ SPTAN1 | aattggatccgcataatggttcacatcaatcag |
| rev_ SPTAN1 | aaatccatgggttctggttcctataaagatccaagcaacct |
| for_ HAVCR1 | aattggatccgcatctttgcgataggtcacatg |
| rev_ HAVCR1 | aaatccatgggttctggttctagcgtgaccctgccat |
| for_ POU4F1 | aattggatccgcggcaccgccgg |
| rev_ POU4F1 | aaatccatgggttctggttccaccagcaccagcaccg |
| for_ PTK6 | aattggatccgcgctcagatacacatcttc |
| rev_ PTK6 | aaatccatgggttctggttcctgctatctggaaagcc |
| for_PRX | aattggatccgccacttctggcaggcg |
| rev_PRX | aaatccatgggttctggttccaaactgccaaaagtgccagaag |

**Table S6. Estimated ROC curves metrics.**

| Markers combination | AUC | ACC | SE | SP |
| --- | --- | --- | --- | --- |
| PRX_451-494_ PTK6_301-344_ | 0.753 | 0.700 | 0.714 | 0.682 |
| SPTAN1_601-644_ PTK6_301-344_ LMP1_285-330_ | 0.774 | 0.740 | 0.714 | 0.773 |
| SPTAN1_601-644_ PRX_451-494_ PTK6_301-344_ LMP1_285-330_ | 0.818 | 0.820 | 0.786 | 0.864 |

Performance of tested analytes as best single and multiple biomarker panels under ROC curve analysis. ROC curve combines accuracy (ACC), sensitivity (SE) and specificity (SP) of a given marker for a diagnostic test from 0.5 (no discriminating power) to 1.0 (complete separation). The combos with high area under curve (AUC), SE and SP were considered as best performing marker ensembles generated from the combinatorial analysis, while those with small SE and SP were considered to be of negligible importance.

References

[1.    Mazzara S, Rossi RL, Grifantini R, Donizetti S, Abrignani S, Bombaci M. CombiROC: an interactive web tool for selecting accurate marker combinations of omics data. Sci Rep. 2017 Mar 30;7:45477.](https://sciwheel.com/work/bibliography/7012284)

[2.    Willis MD, Kreft KL, Dancey B. Oligoclonal bands. Pract Neurol. 2024 Jun 27;](https://sciwheel.com/work/bibliography/16719557)
